# Ontogenetic shifts in brain scaling reflect behavioral changes in the life cycle of the pouched lamprey *Geotria australis*

**DOI:** 10.3389/fnins.2015.00251

**Published:** 2015-07-28

**Authors:** Carlos A. Salas, Kara E. Yopak, Rachael E. Warrington, Nathan S. Hart, Ian C. Potter, Shaun P. Collin

**Affiliations:** ^1^Neuroecology Group, School of Animal Biology and UWA Oceans Institute, The University of Western AustraliaCrawley, WA, Australia; ^2^Centre for Fish and Fisheries Research, School of Veterinary and Life Sciences, Murdoch UniversityMurdoch, WA, Australia

**Keywords:** growth, agnathan, lifestyle, filter feeder, heterochrony, jawless vertebrate, metamorphosis, parasite

## Abstract

Very few studies have described brain scaling in vertebrates throughout ontogeny and none in lampreys, one of the two surviving groups of the early agnathan (jawless) stage in vertebrate evolution. The life cycle of anadromous parasitic lampreys comprises two divergent trophic phases, firstly filter-feeding as larvae in freshwater and secondly parasitism as adults in the sea, with the transition marked by a radical metamorphosis. We characterized the growth of the brain during the life cycle of the pouched lamprey *Geotria australis*, an anadromous parasitic lamprey, focusing on the scaling between brain and body during ontogeny and testing the hypothesis that the vast transitions in behavior and environment are reflected in differences in the scaling and relative size of the major brain subdivisions throughout life. The body and brain mass and the volume of six brain structures of *G. australis*, representing six points of the life cycle, were recorded, ranging from the early larval stage to the final stage of spawning and death. Brain mass does not increase linearly with body mass during the ontogeny of *G. australis*. During metamorphosis, brain mass increases markedly, even though the body mass does not increase, reflecting an overall growth of the brain, with particularly large increases in the volume of the optic tectum and other visual areas of the brain and, to a lesser extent, the olfactory bulbs. These results are consistent with the conclusions that ammocoetes rely predominantly on non-visual and chemosensory signals, while adults rely on both visual and olfactory cues.

## Introduction

Lampreys are extant relatives of an early and diverse group of jawless vertebrates (Kumar and Hedges, 1998; Heimberg et al., 2008; Janvier, 2008; Smith et al., 2013). The results of early studies on the agnathan nervous system (Johnston, 1902; Heier, 1948; Nieuwenhuys, 1977) have thus been used as an indicator of the ancestral condition of the vertebrate brain (Fritzsch and Northcutt, 1993a; Butler and Hodos, 1996; Northcutt, 2002; Gilland and Baker, 2005; Khonsari et al., 2009; Suárez et al., 2014). The design or bauplan of the vertebrate brain and the developmental mechanisms that underlie their subdivisions are considered to be highly conserved (Striedter, 2005; Ota and Kuratani, 2007; Guerin et al., 2009; Charvet et al., 2011). However, it is expected that the various sensory modalities and other neural specializations will evolve, to a degree, in association with ecological niche, and that this relationship will be reflected in adapted behaviors and/or enhanced cognitive capabilities (Barton et al., 1995; Barton and Harvey, 2000; De Winter and Oxnard, 2001). Indeed, brain size and the relative development of major brain subdivisions vary at intraspecific, interspecific, and ontogenetic levels across a range of vertebrates (e.g., Kruska, 2005; Gonda et al., 2013) in relation to factors such as life style, habitat, and behavior (e.g., Pollen et al., 2007; Yopak and Montgomery, 2008; Barton and Capellini, 2011), as well as phylogenetic and developmental constraints (e.g., Finlay and Darlington, 1995; Yopak et al., 2010).

The size of the brain relative to the body (scaling) has long since been used in studies of brain development and evolution (Ariëns Kapper, 1936; Gould, 1975; Deacon, 1990; Aboitiz, 1996), in which brain mass (*E*) is characterized as a function of body mass (*S*) with Snell's formula: *E* = k^*^S^∝^ or log *E* = ∝log *S* + *k*, where ∝ = allometric slope or scaling power. It is a common assumption that encephalization (a larger than expected brain size for a given body size) reflects enhanced cognitive capabilities (Jerison, 1977; Ebbesson, 1980; Lefebvre et al., 2004), although this is still the subject of debate (Healy and Rowe, 2007; Herculano-Houzel, 2012). Previous studies have examined encephalization of the brain of jawless fishes (Platel and Delfini, 1981; Ebinger et al., 1983; Platel and Vesselkin, 1989; Wicht, 1996) and have shown that agnathans, particularly lampreys, possess a relatively small brain and some of the highest degrees of intraspecific variation in brain and body mass when compared to any other vertebrate group (Ebinger et al., 1983; Platel and Delfini, 1986). However, these data have been collected from very few species and no consideration has yet been given to changes in encephalization and brain organization that may occur throughout their life cycle. Indeed, ontogenetic studies of diverse groups of vertebrates have shown that the brain grows at different rates during their lifespan, with the rates being greatest in the embryonic and early postnatal phases (Bauchot et al., 1979; Gille and Salomon, 2000; Fu et al., 2013; Ngwenya et al., 2013). Although some studies have shown shifts in ecology and corresponding shifts in brain development occur in fishes (e.g., Brandstätter and Kotrschal, 1990; Wagner, 2003; Lisney et al., 2007; Iribarne and Castelló, 2014), there are no data on the pattern of encephalization or brain subdivision scaling during the ontogeny of lampreys.

**Figure 1:**
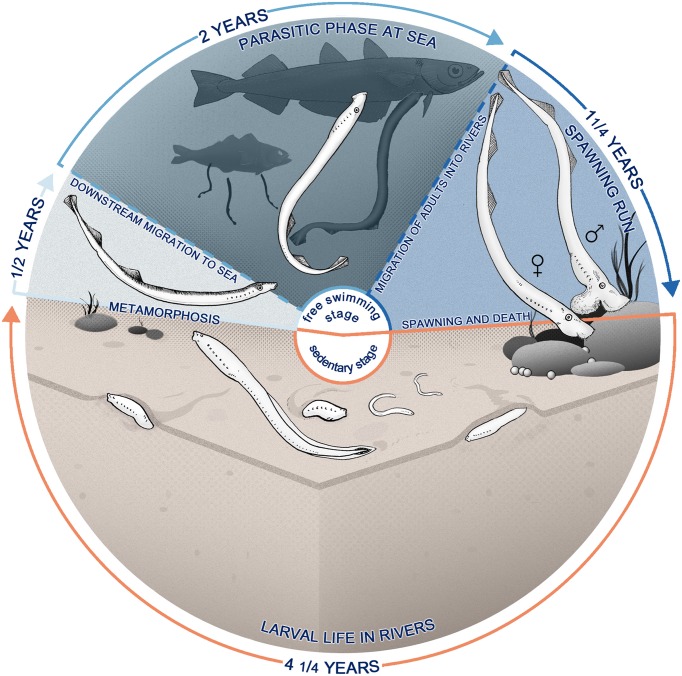
**Life cycle of** ***Geotria australis*** **presenting anadromous reproductive and feeding migrations**. After hatching (**bottom**), the larvae—also called ammocoetes—burrow in the sediments of rivers, becoming a microphagous filter-feeder for approximately 4 years. The larval phase is followed by a metamorphosis, a non-feeding transition to adult stage that lasts for approximately 6 months (**left**), where there is a marked transformation in most of the body systems. Animals at this stage start migrating downstream and enter the sea, where they locate a teleost host and feed on its flesh using a specialized buccal apparatus (**top**). *G. australis* return years later to the rivers, where they start a long upstream migration, subsisting only on body reserves, which are expended in developing secondary sexual characteristics and reproductive behavior. Upstream migrants finally spawn and die (**right**).

The life cycle of lampreys is very conserved (Chang et al., 2014; Potter et al., 2015), consisting of a prolonged and sedentary larval phase, followed by metamorphosis into the free-swimming adult phase (Manzon et al., 2015), as illustrated in Figure 1. In the pouched lamprey *Geotria australis*, which is widely distributed in temperate regions of the southern hemisphere (Renaud, 2011), the life cycle has an approximate duration of 8 years (Potter et al., 1980, 1983; Potter and Hilliard, 1986).

After hatching, the larvae (ammocoetes) burrow in the soft sediments of streams and rivers, filtering detritus, algae and other organisms from the overlying water (Piavis, 1971; Moore and Mallat, 1980; Richardson et al., 2010; Dawson et al., 2015). Ammocoetes have rudimentary eyes with a largely undifferentiated retina (Meyer-Rochow and Stewart, 1996; Villar-Cheda et al., 2008), and also a well-developed non-visual photoreceptive system, e.g., the pineal organ (García-Fernández and Foster, 1994; Deliagina et al., 1995; Melendez-Ferro et al., 2002; Vigh et al., 2002). In fact, they exhibit nocturnal habits with synchronized, seasonal downstream movements (Gritzenko, 1968; Potter, 1980), which may be controlled by circadian rhythms. An octaval lateral line system provide additional mechano-, electro-, and photo-perception, with photoreception being mediated by dermal non-visual photoreceptors located in the tail (Ronan, 1988; Ronan and Bodznick, 1991; Deliagina et al., 1995; Gelman et al., 2007). Ammocoetes also have well-developed gustatory (Baatrup, 1985; Barreiro-Iglesias et al., 2010) and olfactory (Vandenbossche et al., 1995; Zielinski et al., 2005) systems, and behavioral evidence has revealed that rotting potato haulms attracted ammocoetes when placed on the bed of freshwater streams (Enequist, 1937; Hardisty and Potter, 1971), indicating that they may actively search for food using chemosensory cues. Therefore, taste and olfaction are likely important drivers of their behavior.

The metamorphosis of anadromous parasitic species of lampreys, such as *G. australis*, involves major morphological and physiological changes and the development of new sensory and motor capabilities. These include the development of image-forming eyes with the potential for pentachromacy in *G. australis* (Meyer-Rochow and Stewart, 1996; Collin et al., 1999, 2003; Davies et al., 2007), a reduction of lateral line-mediated negative phototaxis that marks a switch from non-visual to visual perception (Binder et al., 2013), the rearrangement of the gustatory and lateral line systems (Currie and Carlsen, 1988; Jørgensen, 2005; Gelman et al., 2008; Barreiro-Iglesias et al., 2010), and the development of a tooth-bearing suctorial disc and “tongue-like” piston with the associated musculature and trigeminal motor innervation (Homma, 1978; Lethbridge and Potter, 1981). Metamorphosis also involves fundamental changes in a number of internal organs, including the intestine and gills, which enable the lamprey to osmoregulate in the sea (Youson et al., 1977; Hilliard et al., 1983; Bartels and Potter, 2004; Reis-Santos et al., 2008).

During the marine parasitic phase, *G. australis* swims toward and attaches to a host, often a teleost fish, and feeds from its flesh (Hilliard et al., 1985; Renaud et al., 2009), thereby increasing in body size from approximately 100 mm and 0.75 g to 620 mm and 220 g (Potter et al., 1980, 1983). There is strong evidence that during its marine parasitic phase, *G. australis* occupies an epipelagic niche in the sea and exhibits diurnal habits (Potter et al., 1979; Cobley, 1996; Collin et al., 1999; Davies et al., 2007). Following the completion of the parasitic phase, the adult lamprey re-enters rivers cued mainly by pheromones that are released by the ammocoetes (Vrieze and Sorensen, 2001; Sorensen et al., 2005; Vrieze et al., 2010, 2011), where they migrate upstream at night (Jellyman et al., 2002; Binder and McDonald, 2007; Vrieze et al., 2011). *Geotria australis* does not feed during its exceptionally long spawning run, using body reserves accumulated during the marine phase to develop secondary sexual characters and mature gonads (Potter et al., 1983; Paton et al., 2011). The life cycle culminates in spawning and subsequent death.

During its life cycle, *G. australis* occupies different ecological niches and encounters diverse environmental conditions, yet there have been no comprehensive studies that have quantified the changes in brain organization corresponding to these marked changes in ecology and behavior. In this study, we assess changes in relative brain size (encephalization) and in the volume of six major brain structures (brain organization) at different phases of the life cycle in *G. australis*. We hypothesize that differences in brain size and organization will reflect the pronounced environmental and physiological changes that lampreys experience during ontogeny.

## Methods

All the procedures described below were performed in accordance with the ethical guidelines of The University of Western Australia Animal Ethics Committee—Research Project RA/3/100/917.

### Data collection

Forty specimens of *G. australis* were analyzed in this study, representing six different points in their life cycle (ammocoetes of second, third, and fourth age class, downstream migrants, upstream migrants, and maturing adults). Specimens within a stage had the same fixation and preservation methods, as shown in Supplementary Table 1, and were captured in the same year (ammocoetes and downstream migrants) or in different years (upstream migrants and maturing adults). Morphometrics (body mass, body length, sex) were collected for each individual when possible. After a period of fixation, the brain was removed from the chondrocranium. The meninges were removed and the cranial nerves were cut to within 0.5 mm of the base. The brains were blotted and weighed to the nearest 0.1 mg (ammocoetes and downstream migrants) or 1 mg (upstream migrants and maturing adults). Neither brain nor body mass were corrected for shrinkage due to fixation.

**Figure 2:**
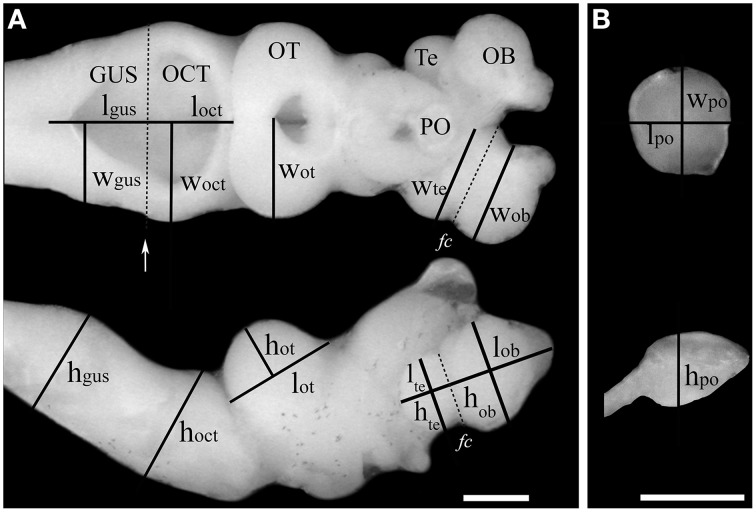
**Estimation of the volume of brain structures using the ellipsoid method**. Measurements of length (l), width (w), and height (h) of six brain structures taken from a dorsal view **(A**, top) or lateral view **(A**, bottom) of the brain of an upstream migrating *G. australis*. In the case of the olfactory bulbs and the telencephalic vesicles, these were defined as parallel or perpendicular lines to the *Fissura circularis* (*fc*), which is highlighted with a discontinuous line in the telencephalon. The limit of the octavo-trigeminal and gustatory areas was defined by a line running parallel to the posterior end of the head of the eighth nerve (white arrow). **(B)** The same measurements were performed in the pineal organ after it was dissected and separated from the remainder of the brain. OB: olfactory bulbs, Te: telencephalic vesicles, PO: pineal organ, OT: optic tectum, OCT: octavo-trigeminal area, Gus: gustatory area. Scale bars = 1 mm.

Photographs of the lateral and dorsal views of each brain were taken using a Leica EC3 camera attached to a Nikon SMZ-745T dissecting microscope. Brains were submerged in a solution of 0.1 M phosphate buffer while photographed to prevent volume distortions caused by dehydration of the tissue. Measurements of length were taken for each of the six brain structures as shown in Figure 2. Brain structures were determined from previously published descriptions of the brain and the cranial nerve distribution in lampreys (Nieuwenhuys and Nicholson, 1998). The length (l), height (h), and width (w) of the olfactory bulbs (OB), telencephalic hemispheres (Te), the pineal organ (PO), the optic tectum (OT), the octaval-trigeminal area (OCT; defined as the anterior region of the rhombencephalon comprising the V–VIII nerves), and the gustatory area (GUS; defined as the posterior region of the rhombencephalon comprising the IX–XII nerves) were measured using ImageJ (Rasband, 1997) as described previously (Huber et al., 1997; Wagner, 2001; Yopak and Lisney, 2012). The pineal organ was dissected out of the brain and photographed separately, see Figure 2B.

Volumes of each major brain structure were estimated using the ellipsoid method, which approximates the volume of a structure by assuming it takes the shape of an idealized ellipsoid, or a fraction of it as shown below (Huber et al., 1997; Wagner, 2001). The general formula of an ellipsoid is:

$$\begin{array}{l} V=\frac{4}{3}\pi \;a\;b\;c \end{array}$$

where *a, b, c* are the radii of the ellipsoid. Using the measurements of length (*l*), height (*h*), and width (*w*) shown for each structure in Figure 2, the volumes were defined as:

$$\begin{array}{l} V=\frac{1}{6}\pi \;l\;h\;w \end{array}$$

for the OB, Te, PO, and the OT, which were all modeled as half ellipsoids,

$$\begin{array}{l} V=\frac{1}{3}\pi \;l\;h\;w \end{array}$$

while the volume of the OCT and GUS were modeled as a quarter of an ellipsoid. In the case of bilateral structures (i.e., OB, Te, and TO), the values of the volumes were doubled. Volume estimates were not corrected for ventricular volume. Total brain volume was calculated from total brain mass using the estimated density of the brain tissue, *d* = 1.036 mg/mm^3^ (Stephan, 1960).

### Age determination

The approximate age of the ammocoete samples was estimated from length-frequency histograms for larval and metamorphosing representatives of *G. australis* (Potter et al., 1980; Potter and Hilliard, 1986). Age of adult stages was inferred from the timing of the upstream migration and sexual maturation (Potter et al., 1983).

### Data analysis

All analyses were performed using the open source software R (R Core Team, 2013). The complete dataset was divided into two subsets, one containing body and brain mass (*n* = 32) and the other containing total brain and brain structure volume estimates (*n* = 39).

#### Linear models

For brain and brain structure scaling analyses, each data set was log_10_ transformed to improve normality prior to analysis, after being multiplied by an arbitrary factor (10 and 1000, respectively), in order to obtain positive values of the variables following log_10_ transformation. We conducted similar analyses on both datasets: we fitted least squares regressions within and between stages, and performed analyses of covariance (ANCOVA), with brain mass as the response variable, body mass as the covariate, and stage as a factor for the brain and body mass comparisons. In the case of the brain structures, total brain structure volume was compared to total brain volume minus total structure volume as a covariate. This was done to account for the bias that exists when a brain subdivision is scaled against total brain mass (which includes the subdivision of interest) (Deacon, 1990; Iwaniuk et al., 2010). To control for similarity within the larval or adult phases of the life cycle, stages were combined in “stage 1” (no combination), “stage 2” (all ammocoetes grouped together), “stage 3” (all adults grouped together), “stage 4” (all ammocoetes grouped together, downstream and upstream migrants grouped together), “stage 5” (all ammocoetes grouped together, upstream migrants and maturing adults grouped together), and “stage 6” (all ammocoetes grouped together, all adults grouped together) (See Supplementary Table 2). Linear models were fitted to each of these factors and the linear assumptions for each were checked using the R package glvla (Pena and Slate, 2014); valid linear models were then compared and selected using the second-order Akaike Information Criterion (AICc); If the best model had a AICc value indistinguishable from the following model(s), they were averaged using multi-model inference methods contained in the R package MuMIn (Barton, 2014), and the relative importance of the factor in the resulting model was used as a criterion for selection. Tukey *Post-hoc* tests were used to detect differences between groups in the selected models.

#### Principal component analysis

We also used a multivariate approach to determine the clustering of the samples in multidimensional space and characterize the patterns of brain organization of *G. australis* at each point of the life cycle. Principal component analysis (PCA) was performed using relative volume of each structure, calculated as a fraction of the sum of the volume of all six brain structures measured within a specimen (Wagner, 2001; Lisney et al., 2007). Structure proportions were normalized using the arcsine square root transformation previous to analysis. PCA was run using the autocovariance matrix and the singular value decomposition method for better numerical accuracy.

## Results

### Brain scaling

**Figure 3:**
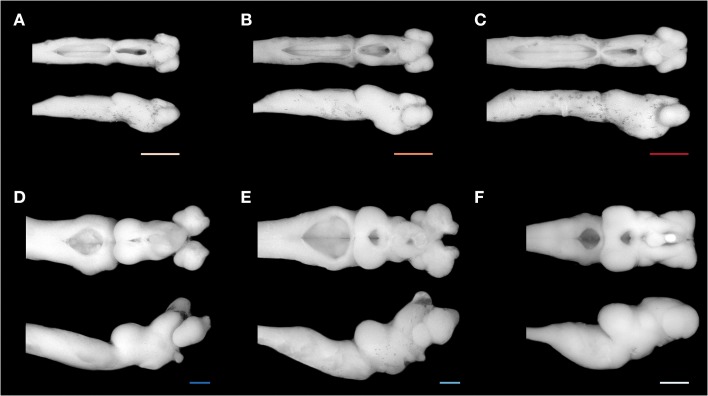
**Brain of** ***Geotria australis*** **during ontogeny**. A representative brain of each stage studied is shown in a dorsal (top) and lateral view (bottom): **(A)** second age class ammocoete, **(B)** third age class ammocoete, **(C)** fourth age class ammocoete, **(D)** maturing adult, **(E)** upstream migrant, and **(F)** downstream migrant. Note the marked difference between the brain of a late ammocoete and a downstream migrant **(C,F)**. Scale bars = 1 mm.

**Figure 4:**
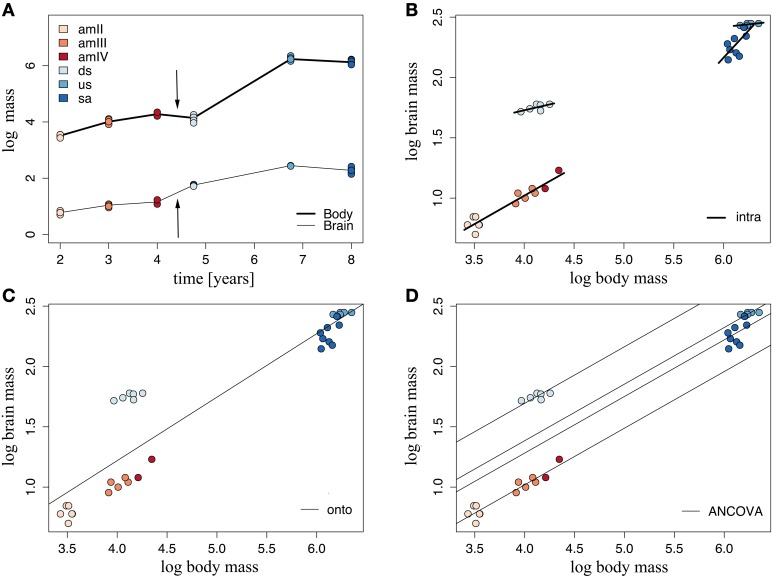
**Brain and body growth vary during the ontogeny of** ***Geotria australis***. **(A)** Brain and body mass growth traced over time. Arrows mark the period of metamorphosis. **(B)** Intraspecific linear regressions, **(C)** Ontogenetic regressions, and **(D)** Linear regressions fitted for each stage after an ANCOVA analysis. For the values of the parameters of these regressions, refer to Supplementary Table 4. amII, second year class ammocoetes; am III, third year class ammocoetes; amIV, fourth year class ammocoetes; ds, downstream migrants; us, upstream migrants; sa, spawning adults.

The brain of *G. australis* shared similar characteristics with those of other species of lampreys (Figure 3) (Wicht, 1996; Nieuwenhuys and Nicholson, 1998). Our analysis of the scaling of brain and body mass in *G. australis* at successive stages of development revealed that the brain and body have different scaling patterns during ontogeny (Figure 4A). Body mass grows at a higher rate than brain mass in both the adult phase and the analyzed period of the larval phase, a trend that is interrupted during metamorphosis (Figure 4A, arrows), where body mass was similar between downstream migrants and the latest ammocoete stage (Two-tailed Welch *t*-test, *T* = 1.98, *p* = 0.201); however, brain mass was significantly higher in downstream migrants as compared to ammocoetes IV (One-tailed Welch *t*-test, *T* = 7.8, *p* = 0.037).

According to the second-order Akaike information criterion, the best model of brain mass as a function of body mass occurred when stage 2 was used as a factor, grouping all ammocoetes together (Supplementary Table 3). We fitted stage-specific (intraspecific) regressions to each of these groups, whose slopes varied across ontogeny (Figure 4B); all groups showed intraspecific negative allometry of brain mass with body mass. The highest rate of brain growth was reached at the larval phase (α = 0.47), followed by downstream and upstream migrants (Supplementary Table 4), while the period of regression of body mass in the course of maturation was accompanied by a steep reduction of brain mass (α = 0.90). We also defined an ontogenetic linear regression as the line of best fit between all specimens, where most of the groups had large deviations from the predicted values of brain mass (Figure 4C), indicating that brain mass does not scale linearly with body mass at all stages in the life cycle of *G. australis*. These two sets of regressions were combined in an analysis of covariance (ANCOVA), the results of which are illustrated in Figure 4D. These data show that both stage 2 and body mass are significant when explaining the observed variance of brain mass (ANCOVA, *p* < 0.001), and no significant interaction between factor (stage 2) and covariate (body mass) is found, indicating no significant differences in the slopes calculated for each group in the stage-specific regressions. The ANCOVA calculated a common slope, with a similar value to the slope obtained in the intraspecific regression of ammocoetes, and different intercepts for each group (See Supplementary Table 4), which represent differences in relative brain mass between groups. The Tukey *Post-hoc* test showed significant differences between all groups of stage 2 (*p* < 0.001); downstream migrants had the highest intercept, demonstrating an increase in relative brain mass at this stage.

### Scaling of brain structures

The analyzed brain structures showed different patterns of growth during the life cycle of *G. australis*. Ontogenetic regressions of total structure volume against total brain volume minus structure volume (hereafter referred to as brain volume) were fitted to each of the structures analyzed and their parameters are tabulated in Supplementary Table 5. A general trend between these regressions was the large deviations from the expected values shown by the downstream migrants, which were positive for the telencephalon and the optic tectum, but negative in the case of the pineal organ, the octavo-trigeminal area and the gustatory area.

**Figure 5:**
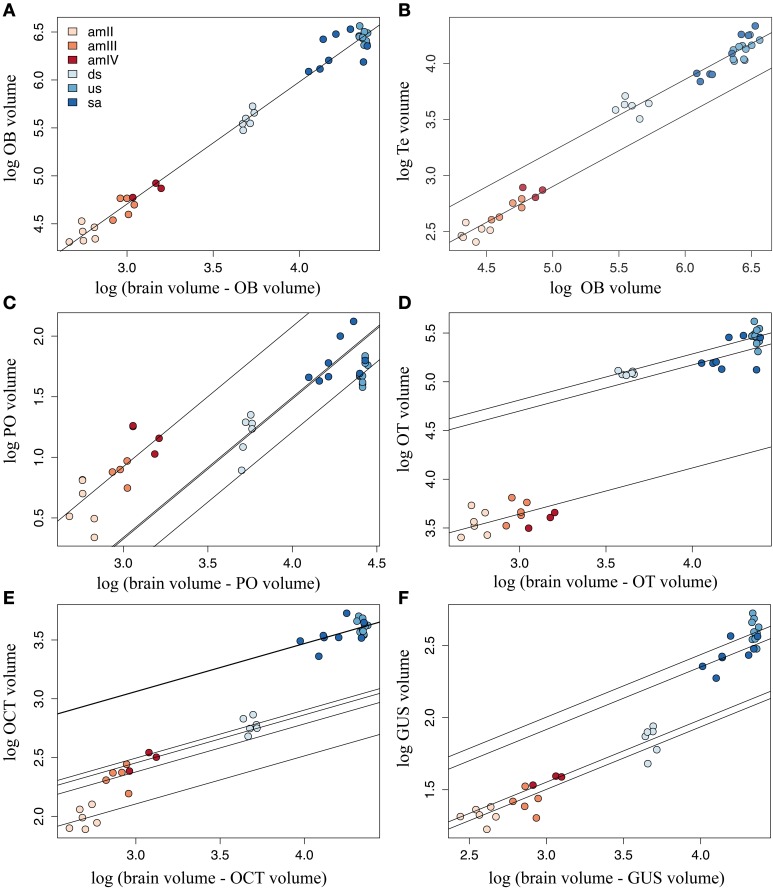
**Calculated regression lines after ANCOVA**. Best linear models are plotted for each structure, showing the differences in scaling of each structure to the rest of the brain: **(A)** olfactory bulbs (OB), **(B)** telencephalic hemispheres (Te), **(C)** pineal organ (PO), **(D)** optic tectum (OT), **(E)** octavo-trigeminal area (OCT), and **(F)** gustatory area (GUS). For the values of the parameters of these models, refer to Supplementary Table 5. amII, second age class ammocoetes; amIII, third age class ammocoetes; amIV, fourth age class ammocoetes; ds, downstream migrants; us, upstream migrants; sa, spawning adults.

The olfactory bulb was the only structure where the observed values fitted the expected values closely in all the stages, supporting a linear scaling of this structure with total brain throughout ontogeny (Figure 5A). Remarkably, the olfactory bulbs showed the steepest hyperallometric growth reported in this study (α = 1.27), generating highly developed olfactory bulbs in upstream migrants and maturing adults. The pineal organ and the octavo-trigeminal area also showed a significant linear fit with total brain volume, as shown in Supplementary Table 5, although this was not the best model for these structures (see below).

Similar to the olfactory bulbs, the telencephalic hemispheres showed a close fit to brain volume in most stages, but because of the high heteroscedasticity in the values of maturing adults, the linear assumptions were violated in this case and in other tested linear models of the telencephalic hemispheres (results not shown). Nevertheless, we found that these assumptions were valid when fitting the telencephalic volume with the volume of the olfactory bulbs, and thus in this case total olfactory bulbs volume was used as covariate in the ANCOVA analysis. The best model for the telencephalic hemispheres included stage 6 as a factor (Figure 5B). This structure showed linear growth with the olfactory bulbs along the larval phase and an increase in size after metamorphosis, which is maintained throughout the adult phase of the life cycle. However, only a marginal difference was detected between ammocoetes and adults (Tukey *Post-hoc* test, *p* = 0.091).

The best models for the pineal organ and the gustatory area had stage 2 as factor, whereas for the octavo-trigeminal area it was stage 1 and for the optic tectum it was stage 4 (Supplementary Table 3). The calculated slope for the pineal organ in the ANCOVA was higher than in the ontogenetic regression, and ammocoetes had the highest intercept (Figure 5C). We found no significant differences between ammocoetes, downstream and upstream migrants, but the pineal organ in maturing adults was significantly different from that of downstream migrants, although only marginally different from upstream migrants (Tukey *Post-hoc* test, *p* = 0.017 and 0.053, respectively). The corrected slope for the optic tectum showed two markedly slow phases of growth, larval and adult, with a significant difference in size between them (Tukey *Post-hoc* test, *p* < 0.05; Figure 5D); the optic tectum of maturing adults was significantly reduced compared to downstream and upstream migrants (Tukey *Post-hoc* test, *p* < 0.05), and not different from the optic tectum of ammocoetes (Tukey *Post-hoc* test, *p* = 0.45).

The volume of the gustatory area of the downstream migrants was significantly different to the other stages (Tukey *Post-hoc* test, *p* < 0.05), with a shallow slope (α = 0.43). However, considering the value of the calculated intercepts in the ANCOVA of the gustatory area, the downstream migrants clustered with ammocoetes, whereas upstream migrants and maturing adults had higher values of intercepts (Figure 5E). This was also the case for the octavo-trigeminal area, where the volume in downstream migrants was different from all the other stages (Tukey *Post-hoc* test, *p* < 0.05) and their volume was closer to ammocoetes than to adults although, in contrast to all other structures, we found that in this area the ammocoetes were best fitted as separate groups, where the second age class ammocoetes had a smaller intercept than other larval stages (Tukey *Post-hoc* tests: amIII, *p* = 0.020; amIV, *p* = 0.083; Figure 5F). Some maturing adults possessed a relatively higher octavo-trigeminal area than upstream migrants, consistent with the modifications of the oral disc and the appearance of the gular sac in this period (Potter et al., 1983; Neira, 1984). However, we did not observed significant differences between these groups. Our results also showed no consistent differences between male and female lampreys in any structure (results not shown).

### Multivariate analysis and stage clustering

**Figure 6:**
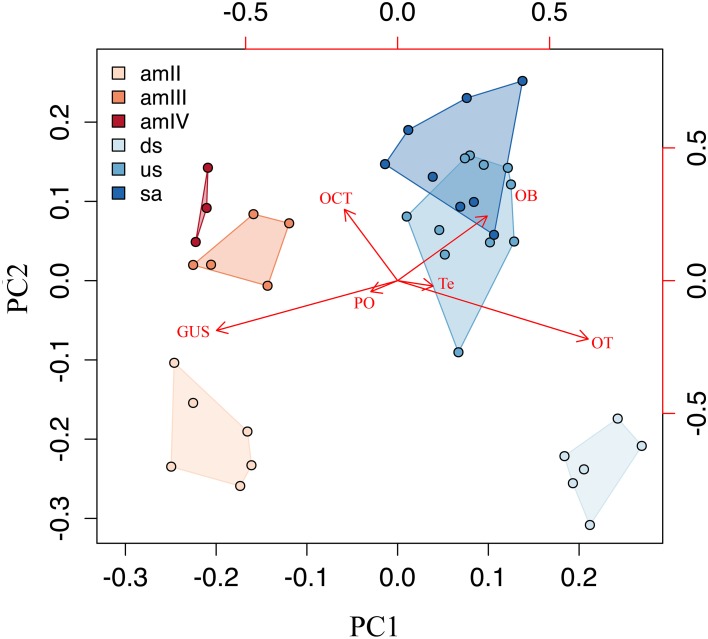
**A scatterplot of principal components PC1 and PC2**. Principal component analysis, representing the major changes in the composition of the brain during the life cycle. OB, olfactory bulbs; Te, telencephalic hemispheres; PO, pineal organ; OT, optic tectum; OCT, octaval-trigeminal area; GUS, gustatory area; amII, second age class ammocoetes; amIII, third age class ammocoetes; amIV, fourth age class ammocoetes; ds, downstream migrants; us, upstream migrants; sa, spawning adults.

The principal component analysis performed on the correlation matrix of the relative size of the six brain structures measured in this study provided a clear separation in the multidimensional space of the two phases of the life cycle of *G. australis*. The relative loadings of the first four components and their relative importance are given in Table 1. The first two components explained 93.3% of the overall variance and their scores are plotted in Figure 6. The first component (PC1) reflects the high loadings for the optic tectum and gustatory area, and secondarily in the olfactory bulbs, separating larvae, which had a relatively large gustatory area and pineal organ, from adults, which had relatively larger optic tecta, olfactory bulbs and telencephalic hemispheres. Similarly, the second component (PC2) separated younger and older individuals within a phase, where older individuals had relatively larger olfactory bulbs and octavo-trigeminal areas than younger individuals in both phases of the life cycle.

**Table 1:** **Results of the principal component analysis for the first four components**.

**Importance of components**	**PC1**	**PC2**	**PC3**	**PC4**
Standard deviation	0.189	0.093	0.046	0.031
Proportion of the variance	0.749	0.183	0.045	0.021
Cumulative proportion	0.749	0.933	0.978	0.998
**RELATIVE LOADINGS**				
OB	0.313	0.523	−0.710	0.150
Te	0.125	−0.044	0.007	−0.868
PO	−0.094	−0.089	0.084	−0.101
TO	0.666	−0.472	0.193	0.345
OCT	−0.186	0.576	0.584	0.217
GUS	−0.633	−0.403	−0.332	0.218

## Discussion

Lampreys experience very different behavioral phases during the life cycle, from a microphagous sedentary mode to an active parasitic mode. This study characterized the growth of the brain (encephalization) during the life cycle of *G. australis*, focusing on the scaling between brain and body throughout ontogeny and testing the hypotheses that the vast transitions in behavior and environment are reflected in differences in both encephalization and the relative development of major brain subdivisions.

The changes occurring in the nervous system of lampreys during ontogeny have attracted the attention of many comparative neurobiologists, who have shown extensive morphological and physiological modifications of the peripheral and central nervous system, such as the development of the visual system (Kennedy and Rubinson, 1977; Kosareva, 1980; De Miguel and Anadon, 1987; Rubinson, 1990; Fritzsch and Northcutt, 1993b; Pombal et al., 1994; Davies et al., 2007; Villar-Cheda et al., 2008). However, in spite of the multiple studies quantifying these changes throughout the life cycle (Tamotsu and Morita, 1986; De Miguel and Anadon, 1987; Currie and Carlsen, 1988; Melendez-Ferro et al., 2003; Vidal Pizarro et al., 2004; Antri et al., 2006), an overall view of the pattern of development of the brain and its organization, including larval and adult phases, has been absent until now.

### Brain scaling

The description of the changes in encephalization during the life cycle of jawless fishes will improve our current understanding brain development at multiple levels. Previous interspecific studies in agnathans have differed on the scaling relationship between brain size and body size of lampreys, ranging from 0.23 (Ebinger et al., 1983) to 0.56 (Platel and Vesselkin, 1988). In addition to discrepancies in the value of the scaling exponent, both studies suffered from low sample sizes, with data on only three (Ebinger et al., 1983) and two (Platel and Vesselkin, 1988) species, out of 41 currently recognized species of lampreys (Potter et al., 2015). This discrepancy in the scaling exponent requires improved resolution, as one value classifies lampreys as being far less encephalized than other gnathostomes, with a slow rate of growth of the brain in relation to the body (α = 0.23), while the other places this group within the known range of the interspecific variation in the scaling exponent between most vertebrate groups (α = 0.56), which usually falls between 0.5 and 0.6 (Striedter, 2005). Similarly, no consensus has been reached with regards to the intraspecific scaling exponent in the sea lamprey *Petromyzon marinus*, which ranges from −0.04 (Ebinger et al., 1983) to 0.56 (Platel and Delfini, 1986). However, given the dramatic shifts that occur throughout the life history of lampreys, these published values for brain scaling are likely to be highly dependent on when in the life cycle the brains were sampled. In fact, this study shows that, as lampreys advance in their upstream migration, they lose both body and brain mass at different rates, which is reflected in a higher intraspecific scaling factor in maturing adults (Figure 4). This variation between early and late upstream migrants may explain previously reported discrepancies in the intraspecific allometric slope in *P. marinus*. Nonetheless, it is possible that the observed differences in relative brain mass may also be related to intraspecific variation between separate populations (Gonda et al., 2011) or according to mating strategies (Kolm et al., 2009), which have also been described in lampreys (Hume et al., 2013).

The ontogenetic scaling of brain and body mass in other basal vertebrate groups, such as teleost fishes, has shown that the larvae of both metamorphic (Bauchot et al., 1979; Tomoda and Uematsu, 1996; Wagner, 2003; Sala et al., 2005) and non-metamorphic fishes (Iribarne and Castelló, 2014) exhibit allometric scaling between brain and body size in the early post-hatching development phase, which may be equivalent to the linear phase of growth reported for ammocoetes in this study. However, in the case of metamorphic fishes, such as the rainbow trout *Oncorhyncus mykiss* or the Japanese eel *Anguilla japonica*, there is no clear evidence of an increase of encephalization associated with metamorphosis (Bauchot et al., 1979; Tomoda and Uematsu, 1996), as our results suggest for lampreys, but constitutes an interesting point that warrants further investigation and should be an area of future study.

Teleost fishes possess continuous growth of both the body and the nervous system throughout life (Bauchot et al., 1979; Leyhausen et al., 1987), as opposed to amniotes where brain growth plateaus before the animal reach its final body size (reviewed in Striedter, 2005), although there are some exceptions (Ngwenya et al., 2013). Yet in lampreys, our results and previous records on *P. marinus* (Ebinger et al., 1983) suggest that, in early upstream migrants (end of the parasitic phase), brain growth may have actually reached a plateau, given the low intraspecific scaling factor found at this point of the life cycle (Figure 4B: α = 0.09 for *G. australis*, α = −0.04 for *P. marinus*), although these values were not statistically significant in either study. In addition, we found evidence that a relative reduction in brain mass occurs in parallel with the typical reduction of body mass in maturing lampreys (Potter et al., 1983; Paton et al., 2011), which has not been previously shown in other ontogenetic studies of brain scaling in vertebrates. Even though complex behavior is generally associated with larger brains (reviewed by Striedter, 2005), lampreys still exhibit sophisticated behaviors, such as nest construction, in this period (Hardisty and Potter, 1971; Sousa et al., 2012; Johnson et al., 2015).

Brain growth in vertebrates has been described as the result of several processes, including cell growth and the addition and elimination of cells (Pirlot and Bernier, 1991; Candal et al., 2005; Bandeira et al., 2009; Fu et al., 2013; Boyd et al., 2015). In lampreys, neuro- and glio-genesis are restricted to ventricular proliferative zones in late embryos and early to mid larval stages (Vidal Pizarro et al., 2004; Villar-Cheda et al., 2006; Guerin et al., 2009) and, although adult neurogenesis is widespread in other basal vertebrate groups (Kaslin et al., 2008), it is considered mostly absent in lampreys (Villar-Cheda et al., 2006; Kempermann, 2012). Taken together, these results suggest that brain growth from late ammocoetes onwards is mainly due to the addition of glia, cell growth, and the establishment of new synapses that contribute to the formation of plexiform tissue or neuropil, as suggested previously for lampreys (Rovainen, 1979, 1996).

### Scaling of brain structures

Transitions in habitat and behavior are common during the development of aquatic vertebrates, even if they do not undergo a metamorphic stage, such as recruitment of fish larvae (Kingsford et al., 2002; Kotrschal et al., 2012; McMenamin and Parichy, 2013) and the use of nursery areas in sharks (e.g., Bethea et al., 2004; Heupel and Simpfendorfer, 2011). Usually these transitions are accompanied by *ad-hoc* sensorimotor specializations (Brandstätter and Kotrschal, 1990; Montgomery and Sutherland, 1997; Lisney et al., 2007; Lecchini et al., 2014). Similarly, adults of both bony and cartilaginous fishes, as well as other vertebrates, possess well-developed adaptations to their ecological niche, which are generally reflected in their nervous system as a variation in the relative size of brain subdivisions (Kotrschal and Palzenberger, 1992; Gonzalez-Voyer et al., 2009; Gonzalez-Voyer and Kolm, 2010; Yopak, 2012). Surprisingly, the relative size of these brain subdivisions appear to be constant between species of parasitic lampreys, despite the diverse aquatic niches in which they inhabit (Renaud, 2011; Potter et al., 2015). We found that the optic tectum and olfactory bulbs in adults of *G. australis* comprise similar proportions of the brain to that of *P. marinus* (Platel and Delfini, 1986) and other species of lampreys (Platel and Vesselkin, 1989), concordant with the lack of appreciable neuroanatomical differences in the brain between lamprey species, as reported previously (Platel and Vesselkin, 1989; Nieuwenhuys and Nicholson, 1998). However, we consider that more species of lampreys needs to be examined, including those with alternative life style strategies, such as parasitic and non-parasitic paired species of lampreys, to have a wider perspective of the diversity found in the nervous system of extant agnathans.

#### Olfactory bulbs

It has been suggested that the level of variation in the relative size of the major brain subdivisions may occur in particular structure in a modular or mosaic fashion (Barton and Harvey, 2000), or with a concerted pattern of allometric scaling (Finlay and Darlington, 1995). It has recently been shown that most major brain areas in cartilaginous fishes scale with a characteristic slope that may be conserved across other vertebrates, including mammals (Yopak et al., 2010). One notable exception is found in the olfactory bulbs, which maintain a level of statistical independence from total brain size in a range of vertebrate groups (Finlay and Darlington, 1995; Gonzalez-Voyer et al., 2009; Yopak et al., 2010, 2015). At the ontogenetic level, however, our analysis of the scaling of the olfactory bulbs shows the opposite pattern, whereby the olfactory bulbs scale very tightly with total brain size, with a highly hyperallometric growth (Figure 5A).

Multiple functional hypotheses have been proposed to explain the relative size of the olfactory bulbs (reviewed in Yopak et al., 2015), including the relationship of olfactory cues with navigation, which may play an important role in lampreys while finding a host or on their way back to rivers for the spawning run (Siefkes et al., 2003; Johnson et al., 2005, 2009; Sorensen et al., 2005; Wagner et al., 2009). The olfactory spatial hypothesis predicts that the size of the olfactory bulbs should covary with navigational ability, which is supported by the olfactory input to the hippocampus (Jacobs, 2012). The statistical independence of the olfactory bulbs is then substantiated by the fact that the olfactory bulbs, the hippocampus, and other associated areas of the telencephalon do not scale as tightly with brain size as do other brain subdivisions (Finlay and Darlington, 1995; Finlay et al., 2001; Gonzalez-Voyer et al., 2009; Yopak et al., 2010) and can vary across mammalian taxa depending on the influence of olfactory cues in their behavior (Reep et al., 2007). If these theories can be applied in the context of the lamprey life cycle, we would therefore expect that, should homologous olfactory areas exist in the telencephalon of *G. australis*, they would also scale isometrically with the rest of the brain in this group during ontogeny. Early descriptions of the telencephalon of the lamprey and later hodological evidence have suggested the presence of a hippocampal primordium or medial pallium (Johnston, 1912; Northcutt and Puzdrowski, 1988; Polenova and Vesselkin, 1993; Northcutt and Wicht, 1997). However, scaling of these telencephalic structures have not been studied in agnathans at any level, and even the existence of a medial pallium is disputed by neuroanatomical descriptions based on molecular markers (Pombal and Puelles, 1999; Weigle and Northcutt, 1999; Pombal et al., 2011). Considering that interspecific scaling of the olfactory bulbs has not yet been described in jawless fishes, the available evidence does not permit any definitive conclusions to be made with regard to differences found in the scaling of the olfactory bulbs between lampreys and other vertebrates.

An alternative explanation of the involvement of olfaction in navigation in lampreys is the hypothesis of dual olfaction, which assumes parallel processing of distinct sets of molecules or environmental odors by the main olfactory system and pheromones by the vomeronasal system, following independent pathways in the brain, and acting synergistically in the regulation of olfactory-guided behaviors (reviewed in Suárez et al., 2012). In lampreys, two anatomically distinct sets of olfactory epithelia have been described that show different patterns of central projections, which suggests the existence of a precursor of the vomeronasal system in this group (Ren et al., 2009; Chang et al., 2013). This accessory olfactory system is tightly coupled to motor areas of the brain, constituting an unusual motor system in vertebrates, which is capable of eliciting swimming movements after olfactory stimulation with both naturally occurring odors and pheromones (Derjean et al., 2010). Since lampreys can detect very low (subpicomolar) concentrations of pheromones (Sorensen et al., 2005), this system may be employed in navigation and other behaviors involving pheromone perception, such as searching for a natal river environment to spawn (Siefkes et al., 2003; Johnson et al., 2005, 2009; Sorensen et al., 2005; Wagner et al., 2009). However, whether these differential central projections vary interspecifically and affect the relative size of the olfactory bulbs and/or a tight coupling between development of the olfactory bulbs and motor areas in the brain is unknown and requires further research.

#### The telencephalic hemispheres

Interspecific studies of the scaling of major brain subdivisions have shown that areas of the brain associated with behavioral and motor complexity, e.g., telencephalon and cerebellum, enlarge disproportionately as brain size increases in a range of vertebrates (Finlay and Darlington, 1995; Finlay et al., 2001; Pollen et al., 2007; Yopak et al., 2010). In lampreys, the everted portion of the telencephalon considered in this study (the cerebral hemispheres or telencephalic hemispheres) can be regarded as the multimodal sensorimotor integration center of the telencephalon, providing a neural substrate for orientation movements of the eyes, trunk, and oral movements, due to direct efferent projections to brainstem motor centers and the optic tectum, in a similar fashion to motor control systems of amniote vertebrates (Ericsson et al., 2013; Grillner and Robertson, 2015; Ocaña et al., 2015). The telencephalic hemispheres are also the main target of secondary olfactory projections from the lateral olfactory bulb, which, in turn, receives its primary afferents from the main olfactory epithelium (Northcutt and Puzdrowski, 1988; Northcutt and Wicht, 1997; Ren et al., 2009; Derjean et al., 2010). Therefore, it is not surprising to find a tight scaling relationship between this structure and the olfactory bulbs (*R*^2^ = 0.987). In addition, this telencephalic area receives afferent fibers from the dorsal thalamus, possibly relaying visual and other sensory input that converge on this thalamic area (Polenova and Vesselkin, 1993; Northcutt and Wicht, 1997). Although not significant, there is some evidence of differences in the size of the telencephalic hemispheres between larvae and adults (Tukey *Post-hoc* test, *p* = 0.091), which may be due to the increase of secondary sensory fibers terminating in this area, as both the primary olfactory system (Vandenbossche et al., 1995; Villar-Cheda et al., 2006) and the primary visual projections to the dorsal thalamus (Kennedy and Rubinson, 1977; Kosareva, 1980) develop during metamorphosis. Despite the various studies on the pallial telencephalon of lampreys, no consensus has been achieved yet in relation to the homology of this area with the pallium of other vertebrates (Northcutt and Puzdrowski, 1988; Nieuwenhuys and Nicholson, 1998; Pombal et al., 2009).

#### The pineal organ

The pineal complex in lampreys is formed by the pineal and the parapineal organs (Eddy and Strahan, 1970; Puzdrowski and Northcutt, 1989; Pombal et al., 1999; Yáñez et al., 1999), which participate in non-visual photo-perception and neuroendocrine control of the circadian rhythms in these animals, as it does in a range of vertebrates (Ekström and Meissl, 1997, 2003; Vernadakis et al., 1998). The pineal organ has also been documented in extinct agnathans, where it was similar in relative size to that of contemporary ammocoetes (Gai et al., 2011), suggesting that non-visual light perception was also highly developed in these extinct groups. The observed morphological and physiological variability of this organ in tetrapods has been linked to latitudinal distribution of the species (Ralph, 1975), nocturnality (Bhatnagar et al., 1986; Haldar and Bishnupuri, 2001), and habitat depth in demersal fishes (Wagner and Mattheus, 2002; Bowmaker and Wagner, 2004), although none of these factors fully explained the variability found in the size and morphology of this organ across species.

The best model for the pineal organ described three distinctive periods of growth in the life cycle of *G. australis*. First, there was consistent hyperallometric growth throughout the larval phase; in the second period, during early adult life, including the marine parasitic phase, we observed that the growth of this organ plateaus after metamorphosis, where the size of the pineal organ of ammocoetes was not significantly different to that of downstream or upstream migrants, opposite to what was observed in the other brain structures; and third, we found a relative increase in the size of the pineal organ during sexual maturation. A similar pattern of growth has been documented in the pineal organ of the arctic lamprey *Lethenteron camtschaticum* (Tamotsu and Morita, 1986). The larval phase and sexual maturation periods anticipate important milestones in the ontogeny of lampreys, such as the onset of metamorphosis and spawning, both of which likely depend on the timing of circadian rhythms (Freamat and Sower, 2013). In this regard, it was shown that metamorphosis was prevented with pinealoctomy in *G. australis* and other species (Eddy and Strahan, 1968; Cole and Youson, 1981), and maturation was delayed in adults of the river lamprey *Lampetra fluviatilis* after the same procedure (Eddy, 1971).

#### The optic tectum

In lampreys and other non-mammalian vertebrates, the optic tectum is the main primary visual center of the brain, receiving extensive topographic retinal (retinotopic) projections to the superficial layers (Butler and Hodos, 1996; Iwahori et al., 1999; De Arriba and Pombal, 2007; Jones et al., 2009). Electroreceptive and other sensory input also converge onto this tectal map (Bodznick and Northcutt, 1981; Ronan and Northcutt, 1990; Robertson et al., 2006), where the relevance of salient stimuli can be assessed, as in other vertebrates (Karamian et al., 1966, 1984; Pombal et al., 2001; Gruberg et al., 2006; Kardamakis et al., 2015), leading to orienting movements of the eye, head and trunk (Saitoh et al., 2007; Ocaña et al., 2015).

Ontogenetic comparisons of the relative size of the optic tectum have been documented in several species of elasmobranchs (Lisney et al., 2007) and teleost fishes (Brandstätter and Kotrschal, 1990; Kotrschal et al., 1990; Wagner, 2003), and have shown a shift from an initially well-developed visual system, followed by a relative reduction in the size of the optic tectum and a corresponding increase in other sensory brain areas, such as those that process olfactory or lateral line input, as the animal matures. This change in brain organization has been associated with shifts in ecological niche, from a well-lit environment in epipelagic fish larvae or nurseries of juvenile elasmobranchs to a different primary habitat as adults. In contrast to these groups, we report an opposite shift in brain organization. In ammocoetes of *G. australis*, the optic tectum underwent moderate growth with total brain size (α = 0.47; Figure 5D). In fact, this structure remains mostly undifferentiated and poorly layered during most of the larval phase in lampreys (Kennedy and Rubinson, 1977; De Miguel and Anadon, 1987; De Miguel et al., 1990) and only the central retina is differentiated (Meyer-Rochow and Stewart, 1996; Villar-Cheda et al., 2008). The major growth of the optic tectum occurs in conjunction with the development of the adult eye, in a rapid process that starts at the end of the larval phase and continues during the initial stages of metamorphosis (Potter et al., 1980; De Miguel and Anadon, 1987). Indeed, it is only at the end of the larval phase that the typical retinotopic projections found in adults reach the optic tectum (Jones et al., 2009; Cornide-Petronio et al., 2011). Soon after metamorphosis (downstream migrants), the relative size of the optic tectum is more similar to that of adults than ammocoetes (Supplementary Table 5, Figure 5D).

This rapid development of the visual system explains the lack of a linear fit of the optic tectum in the ontogenetic scaling of this structure with the rest of the brain. We expect that this fast switch from non-visual to visual perception will also affect the scaling of other visual areas of the brain receiving primary retinal input, such as the dorsal thalamus, and that it may be less pronounced in non-visual areas receiving retinal projections, such as the hypothalamus and pretectal area, which are already developed in ammocoetes, where they participate, for example, in non-visual reflexes (De Miguel and Anadon, 1987; Ullen et al., 1995, 1997; Jones et al., 2009). Nevertheless, the scaling of these visual and non-visual areas of the brain has yet to be studied.

Our results suggest that vision may be important during the parasitic phase, reflected in the high development of the optic tectum during metamorphosis. However, the significant reduction in the size of the optic tectum in maturing adults, which is corroborated with reports of eye degeneration during the spawning run (Applegate, 1950), supports previous evidence that vision is not important in lampreys during their upstream migration (Binder and McDonald, 2007; Johnson et al., 2015).

#### Medulla oblongata

Interspecies comparisons in gnathostomes and agnathans have shown that the size of the rhombencephalon, i.e., the medulla oblongata plus the cerebellum, is well-predicted from total brain size in both groups (Ebinger et al., 1983; Yopak et al., 2010), although in lampreys only cerebellum-like structures can be identified (Weigle and Northcutt, 1998; Northcutt, 2002; Montgomery et al., 2012). When comparing brain subdivisions, the medulla oblongata had the lowest scaling factor in cartilaginous fishes (Yopak et al., 2010), whereas it was the highest in agnathans (Ebinger et al., 1983). Indeed, the medulla accounts for approximately half of the total brain size in adult lampreys (this study, Platel and Vesselkin, 1989), and even more in early larvae (Scott, 1887), although this is not as obvious in downstream migrants (see below). The medulla is the first to develop cranial nerves in lampreys (Kuratani et al., 1997; Barreiro-Iglesias et al., 2008) and maintains a relatively stable scaling relationship with total brain size during the later larval phase and even throughout metamorphosis (Figures 5E, F). However, there was a significant difference in the size of the octavo-trigeminal area between the second-age class ammocoetes and older stages (see intercepts in Supplementary Table 5), which may be related to the development of a number of the diverse sensory and motor systems located in this brain structure, as discussed previously.

The growth of the medulla oblongata during metamorphosis maintains a tight scaling relationship with total brain size in late ammocoetes, which supports previous findings that the motoneurons of the trigeminal nucleus in lampreys are conserved through metamorphosis, in spite of the massive replacement of muscle in the head during this period (Homma, 1978; Rovainen, 1996). This has also been documented in other metamorphic vertebrates, such as frogs (Alley and Omerza, 1998).

However, while several brain structures, e.g., the olfactory bulbs and the optic tectum, exhibit greater rate of growth during metamorphosis, both the octavo-trigeminal and gustatory areas grow with a slower rate during this phase, which is expressed as a lower proportion of this area compared to total brain volume in downstream migrants. Nonetheless, our results show a later growth phase of this subdivision during the parasitic phase, particularly of the octavo-trigeminal area, which may be associated with the development of the musculature of the ventilatory branchial basket and the oropharyngeal region, and to the scaling of other somatic and sensory functions as body size enlarges during the marine parasitic phase (Aboitiz, 1996; Rovainen, 1996; De Winter and Oxnard, 2001).

### Neuroecology of the life cycle

Growth of the central nervous system in lampreys is a discontinuous process, with a variable rate of growth of both total brain and its subdivisions throughout life, which was expressed in the relative size of diverse brain structures in each phase of the life cycle (Figure 6). These patterns of brain organization may be interpreted as “cerebrotypes” (Clark et al., 2001; Iwaniuk and Hurd, 2005; Willemet, 2012, 2013), whereby similar patterns of brain organization exist in species that share certain lifestyle characteristics. In this case, different cerebrotypes may in fact exist within a species at different phases of the life cycle.

The ammocoetes of *G. australis* are less encephalized compared to young adults (downstream migrants), with brains that are characterized by a relatively large gustatory area and a highly developed pineal organ (Figures 3, 6). The relative size of the octavo-trigeminal area is increased in late ammocoetes (Figure 5E), whereas the olfactory bulbs, telencephalic hemispheres and optic tectum were relatively small during the whole larval phase (this study, Scott, 1887). It is possible that these characteristics are related to the requirements of a sessile, burrower lifestyle and/or to filter-feeding specializations in this group. Patterns of brain organization of other filter-feeding vertebrates has been described previously, such as the basking shark *Cetorhinus maximus* and the whale shark *Rhincodon typus* (Kruska, 1988; Yopak and Frank, 2009), and mobulid rays (Ari, 2011), which similarly possess a relatively small telencephalon and mesencephalon (Kruska, 1988; Yopak and Frank, 2009). However, given the drastic differences in the ethology between filter feeding jawless and cartilaginous fishes, it is impossible to draw parallels between patterns of brain organization in these groups. Further research is required to determine the existence of common characteristics in brain organization associated with a filter-feeding lifestyle in lampreys.

In contrast to ammocoetes, adult parasitic lampreys are active swimmers who are highly encephalized and possess a battery of well-developed sensory systems during the adult phase, including vision and olfaction. Correspondingly, they also possess a relatively large telencephalon and olfactory bulbs, structures that may be important in navigation (Derjean et al., 2010; Ocaña et al., 2015), and a relatively large optic tectum, which participates in orientation movements and plays a role in visual processing (Saitoh et al., 2007; Kardamakis et al., 2015). Interestingly, some of these features, such high levels of encephalization and a well-developed optic tectum, have also been observed in many coastal-oceanic and pelagic species of both cartilaginous and bony fishes (Lisney and Collin, 2006; Yopak, 2012; Yopak et al., 2015), which may be related to the sensory requirements of the open water habitat across both jawed and jawless fishes.

## Conclusions

We have employed a widely-used volumetric approach (Huber et al., 1997; Wagner, 2001; Gonzalez-Voyer et al., 2009; Yopak and Lisney, 2012; Lecchini et al., 2014) to quantify differences in the relative size of major brain structures during the ontogeny of lampreys. Our results demonstrate shifts in encephalization between larvae and adults, as well as considerable differences in the relative size of brain subdivisions. Taken together, these shifts in brain organization may reflect the sensory requirements of this species at each stage of the life cycle. The inclusion of data of the growth of the brain and its subdivisions in embryonic, prolarva, and early larval stages of ammocoetes, metamorphic, as well as individuals sampled during the parasitic phase, will provide a more comprehensive insight of the growth of the brain and body during the life cycle of lampreys and eventually allow the use of alternative mathematical functions to describe the process of growth in each phase (i.e., Gompertz models, e.g., Calabrese et al., 2013).

It is yet to be determined whether this pattern of brain development is conserved in other species of lampreys, but we anticipate that it is, based on how conserved the life cycle is in this group (Potter et al., 2015), which could explain the reported homogeneity of the central nervous system between species of lampreys. Further studies on the changes in the brain of lampreys throughout ontogeny will contribute to the understanding of the evolution of the brain in agnathans and across vertebrates.

## Author contributions

SC, NH, IP, and CS contributed to the conception, RW and CS acquired the data, KY and CS designed the analyses and interpreted the data. CS drafted the article, and all authors collaborated in its revision.

### Conflict of interest statement

The Reviewer Thomas Lisney declares that, despite being affiliated to the same institution as authors Carlos A. Salas, Kara E. Yopak, Rachael E. Warrington, Nathan S. Hart and Shaun P. Collin, the review process was handled objectively and no conflict of interest exists. The authors declare that the research was conducted in the absence of any commercial or financial relationships that could be construed as a potential conflict of interest.

## References

[B1] Aboitiz F. (1996). Does bigger mean better? Evolutionary determinants of brain size and structure. Brain Behav. Evol. 47, 225–235. 10.1159/000113243 8724645

[B2] Alley K. E. Omerza F. F. (1998). Reutilization of trigeminal motoneurons during amphibian metamorphosis. Brain Res. 813, 187–190. 10.1016/S0006-8993(98)00980-9 9824695

[B3] Antri M. Cyr A. Auclair F. Dubuc R. (2006). Ontogeny of 5-HT neurons in the brainstem of the lamprey, *Petromyzon marinus*. J. Comp. Neurol. 495, 788–800. 10.1002/cne.20910 16506194

[B4] Applegate V. C. (1950). Natural history of the sea lamprey, *Petromyzon marinus*, in Michigan. U. S. Fish Wildlife Serv. Spec. Sci. Rep. Fish. 55, 1–237. 16202094

[B5] Ari C. (2011). Encephalization and brain organization of mobulid rays (Myliobatiformes, Elasmobranchii) with ecological perspectives. TOANATJ 3, 1–13. 10.2174/1877609401103010001

[B6] Ariëns Kapper J. (1936). Brain-bodyweight relation in human ontogenesis and the “indice de valeur cérébrale” of Anthony and Coupin. Proc. R. Neth. Acad. Arts Sci. 39, 1019–1028.

[B7] Baatrup E. (1985). Physiological studies on the pharyngeal terminal buds in the larval brook lamprey, *Lampetra planeri* (Bloch). Chem. Senses 10, 549–558. 10.1093/chemse/10.4.549

[B8] Bandeira F. Lent R. Herculano-Houzel S. (2009). Changing numbers of neuronal and non-neuronal cells underlie postnatal brain growth in the rat. Proc. Natl. Acad. Sci. U.S.A. 106, 14108–14113. 10.1073/pnas.0804650106 19666520 PMC2729028

[B9] Barreiro-Iglesias A. Anadon R. Rodicio M. C. (2010). The gustatory system of lampreys. Brain Behav. Evol. 75, 241–250. 10.1159/000315151 20664239

[B10] Barreiro-Iglesias A. Villar-Cheda B. Abalo X.-M. Anadón R. Rodicio M. C. (2008). The early scaffold of axon tracts in the brain of a primitive vertebrate, the sea lamprey. Brain. Res. Bull. 75, 42–52. 10.1016/j.brainresbull.2007.07.020 18158094

[B11] Bartels H. Potter I. C. (2004). Cellular composition and ultrastructure of the gill epithelium of larval and adult lampreys: implications for osmoregulation in fresh and seawater. J. Exp. Biol. 207, 3447–3462. 10.1242/jeb.01157 15339941

[B12] Barton K. (2014). MuMIn: Multi-model Inference. R Package Version 1.10.15. Available online at: http://CRAN.R-project.org/package=MuMIn

[B13] Barton R. A. Capellini I. (2011). Maternal investment, life histories, and the costs of brain growth in mammals. Proc. Natl. Acad. Sci. U.S.A. 108, 6169–6174. 10.1073/pnas.1019140108 21444808 PMC3076843

[B14] Barton R. A. Harvey P. H. (2000). Mosaic evolution of brain structure in mammals. Nature 405, 1055–1058. 10.1038/35016580 10890446

[B15] Barton R. A. Purvis A. Harvey P. H. (1995). Evolutionary radiation of visual and olfactory brain systems in primates, bats and insectivores. Philos. Trans. R. Soc. B 348, 381–392. 10.1098/rstb.1995.0076 7480110

[B16] Bauchot R. Diagne M. Ribet J. M. (1979). Post-hatching growth and allometry of the teleost brain. J. Hirnforsch. 20, 29–34. 479575

[B17] Bethea D. M. Buckel J. A. Carlson J. K. (2004). Foraging ecology of the early life stages of four sympatric shark species. Mar. Ecol. Prog. Ser. 268, 245–264. 10.3354/meps268245

[B18] Bhatnagar K. P. Frahm H. D. Stephan H. (1986). The pineal organ of bats: a comparative morphological and volumetric investigation. J. Anat. 147, 143–161. 3693069 PMC1261554

[B19] Binder T. R. McDonald D. G. (2007). Is there a role for vision in the behaviour of sea lampreys (*Petromyzon marinus*) during their upstream spawning migration? Can. J. Fish. Aquat. Sci. 64, 1403–1412. 10.1139/f07-102

[B20] Binder T. R. McDonald D. G. Wilkie M. P. (2013). Reduced dermal photosensitivity in juvenile sea lampreys (*Petromyzon marinus*) reflects life-history-dependent changes in habitat and behaviour. Can. J. Zool. 91, 635–639. 10.1139/cjz-2013-0041

[B21] Bodznick D. Northcutt R. G. (1981). Electroreception in lampreys - evidence that the earliest vertebrates were electroreceptive. Science 212, 465–467. 10.1126/science.7209544 7209544

[B22] Bowmaker J. K. Wagner H.-J. (2004). Pineal organs of deep-sea fish: photopigments and structure. J. Exp. Biol. 207, 2379–2387. 10.1242/jeb.01033 15184510

[B23] Boyd J. L. Skove S. L. Rouanet J. P. Pilaz L.-J. Bepler T. Gordân R.. (2015). Human-chimpanzee differences in a FZD8 enhancer alter cell-cycle dynamics in the developing neocortex. Curr. Biol. 25, 772–779. 10.1016/j.cub.2015.01.041 25702574 PMC4366288

[B24] Brandstätter R. Kotrschal K. (1990). Brain growth patterns in 4 European cyprinid fish species (Cyprinidae, Teleostei): roach (*Rutilus rutilus*), bream (*Abramis brama*), common carp (*Cyprinus carpio*) and sabre carp (*Pelecus cultratus*). Brain Behav. Evol. 35, 195–211. 10.1159/000115867 2379081

[B25] Butler A. B. Hodos W. (1996). Comparative Vertebrate Neuroanatomy: Evolution and Adaptation. New York, NY: Wiley-Liss.

[B26] Calabrese E. Badea A. Watson C. Johnson G. A. (2013). A quantitative magnetic resonance histology atlas of postnatal rat brain development with regional estimates of growth and variability. Neuroimage 71, 196–206. 10.1016/j.neuroimage.2013.01.017 23353030 PMC3639493

[B27] Candal E. Anadon R. Bourrat F. Rodriguez-Moldes I. (2005). Cell proliferation in the developing and adult hindbrain and midbrain of trout and medaka (teleosts): a segmental approach. Brain Res. Dev. Brain. Res. 160, 157–175. 10.1016/j.devbrainres.2005.08.009 16236367

[B28] Chang M.-M. Wu F. Miao D. Zhang J. (2014). Discovery of fossil lamprey larva from the Lower Cretaceous reveals its three-phased life cycle. Proc. Natl. Acad. Sci. U.S.A. 111, 15486–15490. 10.1073/pnas.1415716111 25313060 PMC4217442

[B29] Chang S. Chung-Davidson Y. W. Libants S. V. Nanlohy K. G. Kiupel M. Brown C. T.. (2013). The sea lamprey has a primordial accessory olfactory system. BMC Evol. Biol. 13:172. 10.1186/1471-2148-13-172 23957559 PMC3765145

[B30] Charvet C. J. Striedter G. F. Finlay B. L. (2011). Evo-devo and brain scaling: candidate developmental mechanisms for variation and constancy in vertebrate brain evolution. Brain Behav. Evol. 78, 248–257. 10.1159/000329851 21860220 PMC3221253

[B31] Clark D. A. Mitra P. P. Wang S. S. (2001). Scalable architecture in mammalian brains. Nature 411, 189–193. 10.1038/35075564 11346794

[B32] Cobley N. D. (1996). An observation of live prey capture by black-browed albatross *Diomedea melanophrys*. Mar. Ornithol. 24, 45–46.

[B33] Cole W. C. Youson J. H. (1981). The effect of pinealectomy, continuous light, and continuous darkness on metamorphosis of anadromous sea lampreys, *Petromyzon marinus* L. J. Exp. Biol. 218, 397–404. 10.1002/jez.1402180311 7338724

[B34] Collin S. P. Hart N. S. Shand J. Potter I. C. (2003). Morphology and spectral absorption characteristics of retinal photoreceptors in the southern hemisphere lamprey (*Geotria australis*). Vis. Neurosci. 20, 119–130. 10.1017/S0952523803202030 12916734

[B35] Collin S. P. Potter I. C. Braekevelt C. R. (1999). The ocular morphology of the southern hemisphere lamprey *Geotria australis* gray, with special reference to optical specialisations and the characterisation and phylogeny of photoreceptor types. Brain Behav. Evol. 54, 96–118. 10.1159/000006616 10529522

[B36] Cornide-Petronio M. E. Barreiro-Iglesias A. Anadon R. Rodicio M. C. (2011). Retinotopy of visual projections to the optic tectum and pretectum in larval sea lamprey. Exp. Eye Res. 92, 274–281. 10.1016/j.exer.2011.01.011 21295569

[B37] Currie S. N. Carlsen R. C. (1988). Cranial components of startle behavior in larval and adult lampreys. Neuroscience 24, 709–718. 10.1016/0306-4522(88)90363-6 3362357

[B38] Davies W. L. Cowing J. A. Carvalho L. S. Potter I. C. Trezise A. E. Hunt D. M.. (2007). Functional characterization, tuning, and regulation of visual pigment gene expression in an anadromous lamprey. FASEB J. 21, 2713–2724. 10.1096/fj.06-8057com 17463225

[B39] Dawson H. A. Quintella B. R. Almeida P. R. Treble A. J. Jolley J. C. (2015). The ecology of larval and metamorphosing lampreys, in Lampreys: Biology, Conservation and Control, ed Docker M. F. (New York, NY: Springer), 75–137.

[B40] Deacon T. W. (1990). Problems of ontogeny and phylogeny in brain-size evolution. Int. J. Primatol. 11, 237–282. 10.1007/BF02192870

[B41] De Arriba M. D. Pombal M. A. (2007). Afferent connections of the optic tectum in lampreys: an experimental study. Brain Behav. Evol. 69, 37–68. 10.1159/000095272 16926536

[B42] Deliagina T. G. Ullen F. Gonzalez M. J. Efson H. H. Orlovsky G. N. Grillner S. (1995). Initiation of locomotion by lateral-line photoreceptors in lamprey - behavioral and neurophysiological studies. J. Exp. Biol. 198, 2581–2591. 9320511 10.1242/jeb.198.12.2581

[B43] De Miguel E. Anadon R. (1987). The development of retina and the optic tectum of *Petromyzon marinus*, L. A light microscopic study. J. Hirnforsch. 28, 445–456. 3655334

[B44] De Miguel E. Rodicio C. Anadon R. (1990). Organization of the visual system in larval lampreys: an HRP study. J. Comp. Neurol. 302, 529–542. 10.1002/cne.903020309 1702116

[B45] Derjean D. Moussaddy A. Atallah E. St-Pierre M. Auclair F. Chang S.. (2010). A novel neural substrate for the transformation of olfactory inputs into motor output. PLoS Biol. 8:e1000567. 10.1371/journal.pbio.1000567 21203583 PMC3006349

[B46] De Winter W. Oxnard C. E. (2001). Evolutionary radiations and convergences in the structural organization of mammalian brains. Nature 409, 710–714. 10.1038/35055547 11217859

[B47] Ebbesson S. E. (1980). The parcellation theory and its relation to interspecific variability in brain organization, evolutionary and ontogenetic development, and neuronal plasticity. Cell Tissue Res. 213, 179–212. 10.1007/BF00234781 7459999

[B48] Ebinger P. Wächtler K. Stähler S. (1983). Allometrical studies in the brain of cyclostomes. J. Hirnforsch. 24, 545–550. 6663054

[B49] Eddy J. M. P. (1971). The pineal complex, in The Biology of Lampreys, eds Hardisty M. W. Potter I. C. (London: Academic Press), 91–103.

[B50] Eddy J. M. P. Strahan R. (1968). The role of the pineal complex in the pigmentary effector system of the lampreys, *Mordacia mordax* (Richardson) and *Geotria australis* (Gray). Gen. Comp. Endocrinol. 11, 528–534. 10.1016/0016-6480(68)90067-1 5726272

[B51] Eddy J. M. P. Strahan R. (1970). The structure of the epiphyseal complex of *Mordacia mordax* and *Geotria australis* (Petromyzonidae). Acta Zool. 51, 67–84. 10.1111/j.1463-6395.1970.tb00418.x

[B52] Ekström P. Meissl H. (1997). The pineal organ of teleost fishes. Rev. Fish. Biol. Fish. 7, 199–284. 10.1023/A:1018483627058

[B53] Ekström P. Meissl H. (2003). Evolution of photosensory pineal organs in new light: the fate of neuroendocrine photoreceptors. Philos. Trans. R. Soc. Lond. B 358, 1679–1700. 10.1098/rstb.2003.1303 14561326 PMC1693265

[B54] Enequist P. (1937). Das bachneunauge als ökologische modifikation des flussneunauges. Über die fluss – und bachneunaugen schwedens; vorläufige mitteilung. Ark. Zool. 29, 1–22.

[B55] Ericsson J. Stephenson-Jones M. Kardamakis A. Robertson B. Silberberg G. Grillner S. (2013). Evolutionarily conserved differences in pallial and thalamic short-term synaptic plasticity in striatum. J. Physiol. 591, 859–874. 10.1113/jphysiol.2012.236869 23148315 PMC3591703

[B56] Finlay B. L. Darlington R. B. (1995). Linked regularities in the development and evolution of mammalian brains. Science 268, 1578–1584. 10.1126/science.7777856 7777856

[B57] Finlay B. L. Darlington R. B. Nicastro N. (2001). Developmental structure in brain evolution. Behav. Brain Sciences 24, 263–278. 10.1017/S0140525X01003958 11530543

[B58] Freamat M. Sower S. A. (2013). Integrative neuro-endocrine pathways in the control of reproduction in lamprey: a brief review. Front. Endocrinol. 4:151. 10.3389/fendo.2013.00151 24151489 PMC3798812

[B59] Fritzsch B. Northcutt R. G. (1993a). Cranial and spinal nerve organization in amphioxus and lampreys: evidence for an ancestral craniate pattern. Acta Anat. 148, 96–109. 10.1159/000147529 8109201

[B60] Fritzsch B. Northcutt R. G. (1993b). Origin and migration of trochlear, oculomotor and abducent motor neurons in *Petromyzon marinus* L. Brain Res. Dev. Brain Res. 74, 122–126. 10.1016/0165-3806(93)90091-N 8403365

[B61] Fu Y. Rusznak Z. Herculano-Houzel S. Watson C. Paxinos G. (2013). Cellular composition characterizing postnatal development and maturation of the mouse brain and spinal cord. Brain Struct. Funct. 218, 1337–1354. 10.1007/s00429-012-0462-x 23052551

[B62] Gai Z. K. Donoghue P. C. J. Zhu M. Janvier P. Stampanoni M. (2011). Fossil jawless fish from China foreshadows early jawed vertebrate anatomy. Nature 476, 324–327. 10.1038/nature10276 21850106

[B63] García-Fernández J. M. Foster R. G. (1994). Immunocytochemical identification of photoreceptor proteins in hypothalamic cerebrospinal fluid-contacting neurons of the larval lamprey (*Petromyzon marinus*). Cell Tissue Res. 275, 319–326. 10.1007/BF00319430

[B64] Gelman S. Ayali A. Kiemel T. Sanovich E. Cohen A. H. (2008). Metamorphosis-related changes in the lateral line system of lampreys, *Petromyzon marinus*. J. Comp. Physiol. A. 194, 945–956. 10.1007/s00359-008-0367-6 18795304

[B65] Gelman S. Ayali A. Tytell E. D. Cohen A. H. (2007). Larval lampreys possess a functional lateral line system. J. Comp. Physiol. A 193, 271–277. 10.1007/s00359-006-0183-9 17119976

[B66] Gilland E. Baker R. (2005). Evolutionary patterns of cranial nerve efferent nuclei in vertebrates. Brain Behav. Evol. 66, 234–254. 10.1159/000088128 16254413

[B67] Gille U. Salomon F. V. (2000). Brain growth in mallards, Pekin and Muscovy ducks (Anatidae). J. Zool. 252, 399–404. 10.1111/j.1469-7998.2000.tb00635.x

[B68] Gonda A. Herczeg G. Merila J. (2011). Population variation in brain size of nine-spined sticklebacks (*Pungitius pungitius*)–local adaptation or environmentally induced variation? BMC Evol. Biol. 11:75. 10.1186/1471-2148-11-75 21435215 PMC3072340

[B69] Gonda A. Herczeg G. Merilä J. (2013). Evolutionary ecology of intraspecific brain size variation: a review. Ecol. Evol. 3, 2751–2764. 10.1002/ece3.627 24567837 PMC3930043

[B70] Gonzalez-Voyer A. Kolm N. (2010). Sex, ecology and the brain: evolutionary correlates of brain structure volumes in *Tanganyikan cichlids*. PLoS ONE 5:e14355. 10.1371/journal.pone.0014355 21179407 PMC3003682

[B71] Gonzalez-Voyer A. Winberg S. Kolm N. (2009). Brain structure evolution in a basal vertebrate clade: evidence from phylogenetic comparative analysis of cichlid fishes. BMC Evol. Biol. 9:238. 10.1186/1471-2148-9-238 19772561 PMC2755010

[B72] Gould S. J. (1975). Allometry in primates, with emphasis on scaling and the evolution of the brain. Contrib. Primatol. 5, 244–292. 803425

[B73] Grillner S. Robertson B. (2015). The basal ganglia downstream control of brainstem motor centres—an evolutionarily conserved strategy. Curr. Opin. Neurobiol. 33, 47–52. 10.1016/j.conb.2015.01.019 25682058

[B74] Gritzenko O. F. (1968). On the question of an ecological paralIelism between lampreys and salmon (in Russian). Izv. Tikhookean. Nauchno-lssled. Inst. Rybn. Khoz. Okeanogr. 65, 157–169.

[B75] Gruberg E. Dudkin E. Wang Y. Marin G. Salas C. Sentis E.. (2006). Influencing and interpreting visual input: the role of a visual feedback system. J. Neurosci. 26, 10368–10371. 10.1523/JNEUROSCI.3288-06.2006 17035519 PMC6674696

[B76] Guerin A. D'aubenton-Carafa Y. Marrakchi E. Da Silva C. Wincker P. Mazan S.. (2009). Neurodevelopment genes in lampreys reveal trends for forebrain evolution in craniates. PLoS ONE 4:e5374. 10.1371/journal.pone.0005374 19399187 PMC2671401

[B77] Haldar C. Bishnupuri K. S. (2001). Comparative view of pineal gland morphology of nocturnal and diurnal birds of tropical origin. Microsc. Res. Tech. 53, 25–32. 10.1002/jemt.1065 11279667

[B78] Hardisty M. W. Potter I. C. (1971). The Biology of Lampreys. London: AcademicPress.

[B79] Healy S. D. Rowe C. (2007). A critique of comparative studies of brain size. Proc. R. Soc. Lond. B 274, 453–464. 10.1098/rspb.2006.3748 17476764 PMC1766390

[B80] Heier P. (1948). Fundamental principles in the structure of the brain of *Petromyzon fluviatilis*. Acta Anat. 5, 1–213.18876830

[B81] Heimberg A. M. Sempere L. F. Moy V. N. Donoghue P. C. J. Peterson K. J. (2008). MicroRNAs and the advent of vertebrate morphological complexity. Proc. Natl. Acad. Sci. U.S.A. 105, 2946–2950. 10.1073/pnas.0712259105 18287013 PMC2268565

[B82] Herculano-Houzel S. (2012). The remarkable, yet not extraordinary, human brain as a scaled-up primate brain and its associated cost. Proc. Natl. Acad. Sci. U.S.A. 109, 10661–10668. 10.1073/pnas.1201895109 22723358 PMC3386878

[B83] Heupel M. R. Simpfendorfer C. A. (2011). Estuarine nursery areas provide a low-mortality environment for young bull sharks Carcharhinus leucas. Mar. Ecol. Prog. Ser. 433, 237–244. 10.3354/meps09191

[B84] Hilliard R. W. Bird D. J. Potter I. C. (1983). Metamorphic changes in the intestine of three species of lampreys. J. Morphol. 176, 181–196. 10.1002/jmor.1051760207 30060636

[B85] Hilliard R. W. Potter I. C. Macey D. J. (1985). The dentition and feeding mechanisms in adults of the Southern Hemisphere lamprey *Geotria australis* Gray. Acta Zool. 66, 159–170. 10.1111/j.1463-6395.1985.tb00834.x

[B86] Homma S. (1978). Organization of the trigeminal motor nucleus before and after metamorphosis in lampreys. Brain Res. 140, 33–42. 10.1016/0006-8993(78)90236-6 626885

[B87] Huber R. van Staaden M. J. Kaufman L. S. Liem K. F. (1997). Microhabitat use, trophic patterns, and the evolution of brain structure in African cichlids. Brain Behav. Evol. 50, 167–182. 10.1159/000113330 9288416

[B88] Hume J. B. Adams C. E. Mable B. Bean C. W. (2013). Sneak male mating tactics between lampreys (*Petromyzontiformes*) exhibiting alternative life-history strategies. J. Fish Biol. 82, 1093–1100. 10.1111/jfb.12047 23464566

[B89] Iribarne L. Castelló M. E. (2014). Postnatal brain development of the pulse type, weakly electric gymnotid fish *Gymnotus omarorum*. J. Physiol. Paris 108, 47–60. 10.1016/j.jphysparis.2014.05.001 24844821

[B90] Iwahori N. Kawawaki T. Baba J. (1999). Neuronal organization of the optic tectum in the river lamprey, *Lampetra japonica*: a Golgi study. J. Hirnforsch. 39, 409–424. 10536874

[B91] Iwaniuk A. N. Gutierrez-Ibanez C. Pakan J. M. Wylie D. R. (2010). Allometric scaling of the tectofugal pathway in birds. Brain Behav. Evol. 75, 122–137. 10.1159/000311729 20516660

[B92] Iwaniuk A. N. Hurd P. L. (2005). The evolution of cerebrotypes in birds. Brain Behav. Evol. 65, 215–230. 10.1159/000084313 15761215

[B93] Jacobs L. F. (2012). From chemotaxis to the cognitive map: the function of olfaction. Proc. Natl. Acad. Sci. U.S.A. 109, 10693–10700. 10.1073/pnas.1201880109 22723365 PMC3386877

[B94] Janvier P. (2008). Early jawless vertebrates and cyclostome origins. Zoolog. Sci. 25, 1045–1056. 10.2108/zsj.25.1045 19267641

[B95] Jellyman D. J. Glova G. J. Sykes J. R. E. (2002). Movements and habitats of adult lamprey (*Geotria australis)* in two New Zealand waterways. N. Z. J. Mar. Freshwatwer. Res. 36, 53–65. 10.1080/00288330.2002.9517070

[B96] Jerison H. J. (1977). The theory of encephalization. Ann. N. Y. Acad. Sci. 299, 146–160. 10.1111/j.1749-6632.1977.tb41903.x 280197

[B97] Johnson N. S. Buchinger T. J. Li W. (2015). Reproductive ecology of lampreys, in Lampreys: Biology, Conservation and Control, ed Docker M. F. (New York, NY: Springer), 265–303.

[B98] Johnson N. S. Siefkes M. J. Li W. (2005). Capture of ovulating female sea lampreys in traps baited with spermiating male sea lampreys. North Am. J. Fish. Manage. 25, 67–72. 10.1577/M03-226.1

[B99] Johnson N. S. Yun S. S. Thompson H. T. Brant C. O. Li W. (2009). A synthesized pheromone induces upstream movement in female sea lamprey and summons them into traps. Proc. Natl. Acad. Sci. U.S.A. 106, 1021–1026. 10.1073/pnas.0808530106 19164592 PMC2633527

[B100] Johnston J. B. (1902). The brain of Petromyzon. J. Comp. Neurol. 12, 1–86. 10.1002/cne.910120102

[B101] Johnston J. B. (1912). The telencephalon in cyclostomes. J. Comp. Neurol. 22, 341–404. 10.1002/cne.900220401

[B102] Jones M. R. Grillner S. Robertson B. (2009). Selective projection patterns from subtypes of retinal ganglion cells to tectum and pretectum: distribution and relation to behavior. J. Comp. Neurol. 517, 257–275. 10.1002/cne.22154 19760658

[B103] Jørgensen J. (2005). Morphology of electroreceptive sensory organs, in Electroreception, eds Bullock T. Hopkins C. Popper A. Fay R. (New York, NY: Springer), 47–67.

[B104] Karamian A. I. Vesselkin N. P. Belekhova M. G. Zagorulko T. M. (1966). Electrophysiological characteristics of tectal and thalamo-cortical divisions of the visual system in lower vertebrates. J. Comp. Neurol. 127, 559–576. 10.1002/cne.901270408 5968991

[B105] Karamian A. I. Vesselkin N. P. Agayan A. L. (1984). Electrophysiological and behavioral studies of the optic tectum in cyclostomes, in Comparative Neurology of the Optic Tectum, ed Vanegas H. (New York, NY: Plenum Press), 15–30.

[B106] Kardamakis A. A. Saitoh K. Grillner S. (2015). Tectal microcircuit generating visual selection commands on gaze-controlling neurons. Proc. Natl. Acad. Sci. U.S.A. 112, E1956–E1965. 10.1073/pnas.1504866112 25825743 PMC4403191

[B107] Kaslin J. Ganz J. Brand M. (2008). Proliferation, neurogenesis and regeneration in the non-mammalian vertebrate brain. Philos. Trans. R. Soc. Lond. B Biol. Sci. 363, 101–122. 10.1098/rstb.2006.2015 17282988 PMC2605489

[B108] Kempermann G. (2012). New neurons for ‘survival of the fittest.’ Nat. Rev. Neurosci. 13, 727–736. 10.1038/nrn3319 22948073

[B109] Kennedy M. C. Rubinson K. (1977). Retinal projections in larval, transforming and adult sea lamprey, *Petromyzon marinus*. J. Comp. Neurol. 171, 465–479. 10.1002/cne.901710404 833354

[B110] Khonsari R. H. Li B. Vernier P. Northcutt R. G. Janvier P. (2009). Agnathan brain anatomy and craniate phylogeny. Acta Zool. 90, 52–68. 10.1111/j.1463-6395.2008.00388.x

[B111] Kingsford M. J. Leis J. M. Shanks A. Lindeman K. C. Morgan S. G. Pineda J. (2002). Sensory environments, larval abilities and local self-recruitment. Bull. Mar. Sci. 70, 309–340.

[B112] Kolm N. Gonzalez-Voyer A. Brelin D. Winberg S. (2009). Evidence for small scale variation in the vertebrate brain: mating strategy and sex affect brain size and structure in wild brown trout (*Salmo trutta*). J. Evol. Biol. 22, 2524–2531. 10.1111/j.1420-9101.2009.01875.x 19878498

[B113] Kosareva A. A. (1980). Retinal projections in lamprey (*Lampetra fluviatilis*). J. Hirnforsch. 21, 243–256. 6158536

[B114] Kotrschal A. Heckel G. Bonfils D. Taborsky B. (2012). Life-stage specific environments in a cichlid fish: implications for inducible maternal effects. Evol. Ecol. 26, 123–137. 10.1007/s10682-011-9495-5

[B115] Kotrschal K. Adam H. Brandstätter R. Junger H. Zaunreiter M. Goldschmid A. (1990). Larval size constraints determine directional ontogenetic shifts in the visual system of teleosts. J. Zoolog. Syst. Evol. Res. 28, 166–182. 10.1111/j.1439-0469.1990.tb00374.x

[B116] Kotrschal K. Palzenberger M. (1992). Neuroecology of cyprinids: comparative, quantitative histology reveals diverse brain patterns. Environ. Biol. Fish. 33, 135–152. 10.1007/BF00002560

[B117] Kruska D. C. T. (1988). The brain of the basking shark (*Cetorhinus maximus)*. Brain Behav. Evol. 32, 353–363. 10.1159/000116562 3228691

[B118] Kruska D. C. T. (2005). On the evolutionary significance of encephalization in some eutherian mammals: effects of adaptive radiation, domestication, and feralization. Brain Behav. Evol. 65, 73–108. 10.1159/000082979 15627722

[B119] Kumar S. Hedges S. B. (1998). A molecular timescale for vertebrate evolution. Nature 392, 917–920. 10.1038/31927 9582070

[B120] Kuratani S. Ueki T. Aizawa S. Hirano S. (1997). Peripheral development of cranial nerves in a cyclostome, *Lampetra japonica*: morphological distribution of nerve branches and the vertebrate body plan. J. Comp. Neurol. 384, 483–500. 9259485

[B121] Lecchini D. Lecellier G. Lanyon R. G. Holles S. Poucet B. Duran E. (2014). Variation in brain organization of coral reef fish larvae according to life history traits. Brain Behav. Evol. 83, 17–30. 10.1159/000356787 24401605

[B122] Lefebvre L. Reader S. M. Sol D. (2004). Brains, innovations and evolution in birds and primates. Brain Behav. Evol. 63, 233–246. 10.1159/000076784 15084816

[B123] Lethbridge R. C. Potter I. C. (1981). The development of teeth and associated feeding structures during the metamorphosis of the lamprey, *Geotria australis*. Acta Zool. 62, 201–214. 10.1111/j.1463-6395.1981.tb00629.x

[B124] Leyhausen C. Kirschbaum F. Szabo T. Erdelen M. (1987). Differential growth in the brain of the weakly electric fish, *Apteronotus leptorhynchus* (Gymnotiformes), during ontogeny. Brain Behav. Evol. 30, 230–248. 10.1159/000118648 3664264

[B125] Lisney T. J. Bennett M. B. Collin S. P. (2007). Volumetric analysis of the sensory brain areas indicates ontogenetic shifts in the relative importance of sensory systems in elasmobranchs. Raff. Bull. Zool. 14, 7–15. 10.1111/j.0022-1112.2006.00940.x

[B126] Lisney T. J. Collin S. P. (2006). Brain morphology in large pelagic fishes: a comparison between sharks and teleosts. J. Fish Biol. 68, 532–554. 10.1111/j.0022-1112.2006.00940.x

[B127] Manzon R. G. Youson J. H. Holmes J. A. (2015). Lamprey metamorphosis, in Lampreys: Biology, Conservation and Control, ed Docker M. F. (New York, NY: Springer), 139–214.

[B128] McMenamin S. K. Parichy D. M. (2013). Metamorphosis in teleosts. Curr. Top. Dev. Biol. 103, 127–165. 10.1016/b978-0-12-385979-2.00005-8 23347518 PMC5606158

[B129] Melendez-Ferro M. Perez-Costas E. Villar-Cheda B. Rodriguez-Munoz R. Anadon R. Rodicio M. C. (2003). Ontogeny of gamma-aminobutyric acid-immunoreactive neurons in the rhombencephalon and spinal cord of the sea lamprey. J. Comp. Neurol. 464, 17–35. 10.1002/cne.10773 12866126

[B130] Melendez-Ferro M. Villar-Cheda B. Abalo X. M. Perez-Costas E. Rodriguez-Munoz R. Degrip W. J.. (2002). Early development of the retina and pineal complex in the sea lamprey: comparative immunocytochemical study. J. Comp. Neurol. 442, 250–265. 10.1002/cne.10090 11774340

[B131] Meyer-Rochow V. B. Stewart D. (1996). Review of larval and postlarval eye ultrastructure in the lamprey (Cyclostomata) with special emphasis on *Geotria australis* (Gray). Microsc. Res. Tech. 35, 431–444. 9016447 10.1002/(SICI)1097-0029(19961215)35:6<431::AID-JEMT3>3.0.CO;2-L

[B132] Montgomery J. C. Bodznick D. Yopak K. E. (2012). The cerebellum and cerebellum-like structures of cartilaginous fishes. Brain Behav. Evol. 80, 152–165. 10.1159/000339868 22986830

[B133] Montgomery J. C. Sutherland K. B. W. (1997). Sensory development of the antarctic silverfish *Pleuragramma antarcticum*: a test for the ontogenetic shift hypothesis. Polar Biol. 18, 112–115. 10.1007/s003000050165

[B134] Moore J. W. Mallat J. M. (1980). Feeding of larval lamprey. Can. J. Fish. Aquat. Sci. 37, 1658–1664. 10.1139/f80-213

[B135] Neira F. J. (1984). Biomorfología de las lampreas parasitarias chilenas *Geotria australis* (Gray, 1851) y *Mordacia lapicida* (Gray, 1851)(Petromyzontiformes). Gayana Zool. 48, 3–40.

[B136] Ngwenya A. Patzke N. Spocter M. A. Kruger J.-L. Dell L.-A. Chawana R.. (2013). The continuously growing central nervous system of the Nile crocodile (*Crocodylus niloticus*). Anat. Rec. 296, 1489–1500. 10.1002/ar.22752 23832836

[B137] Nieuwenhuys R. (1977). The brain of the lamprey in a comparative perspective. Ann. N. Y. Acad. Sci. 299, 97–145. 10.1111/j.1749-6632.1977.tb41902.x 280225

[B138] Nieuwenhuys R. Nicholson C. (1998). Lampreys, Petromyzontoidae, in The Central Nervous System of Vertebrates, eds Nieuwenhuys R. Donkelaar H. J. T. Nicholson C. (Berlin: Springer), 397–495. 10.1007/978-3-642-18262-4_10

[B139] Northcutt R. G. (2002). Understanding vertebrate brain evolution. Integr. Comp. Biol. 42, 743–756. 10.1093/icb/42.4.743 21708771

[B140] Northcutt R. G. Puzdrowski R. L. (1988). Projections of the olfactory bulb and nervus terminalis in the silver lamprey. Brain Behav. Evol. 32, 96–107. 10.1159/000116537 3179698

[B141] Northcutt R. G. Wicht H. (1997). Afferent and efferent connections of the lateral and medial pallia of the silver lamprey. Brain Behav. Evol. 49, 1–19. 10.1159/000112978 8980849

[B142] Ocaña F. M. Suryanarayana S. M. Saitoh K. Kardamakis A. A. Capantini L. Robertson B.. (2015). The lamprey pallium provides a blueprint of the mammalian motor projections from cortex. Curr. Biol. 25, 413–423. 10.1016/j.cub.2014.12.013 25619762

[B143] Ota K. G. Kuratani S. (2007). Cyclostome embryology and early evolutionary history of vertebrates. Integr. Comp. Biol. 47, 329–337. 10.1093/icb/icm022 21672842

[B144] Paton K. R. Cake M. H. Potter I. C. (2011). Metabolic responses to exhaustive exercise change markedly during the protracted non-trophic spawning migration of the lamprey *Geotria australis*. J. Comp. Physiol. B 181, 751–763. 10.1007/s00360-011-0570-6 21442322

[B145] Pena E. A. Slate E. H. (2014). gvlma: Global Validation of Linear Models Assumptions. R Package Version 1.0.0.2. Available online at: http://CRAN.R-project.org/package=gvlma

[B146] Piavis G. W. (1971). Embriology, in The Biology of Lampreys, eds Hardisty M. W. Potter I. C. (London: Academic Press), 361–400.

[B147] Pirlot P. Bernier R. (1991). Brain growth and differentiation in two fetal bats: qualitative and quantitative aspects. Am. J. Anat. 190, 167–181. 10.1002/aja.1001900206 2012004

[B148] Platel R. Delfini C. (1981). L'Encéphalisation chez la myxine (*Myxine glutinosa* L.). Analyse quantifiée des principales subdivisions encéphaliques. Cah. Biol. Mar. 22, 407–430. 3760543

[B149] Platel R. Delfini C. (1986). L'Encéphalisation chez la lamproie marine, *Petromyzon marinus* (L.). Analyse quantifiée des principales subdivisions encéphaliques. J. Hirnforsch. 3, 279–293. 3760543

[B150] Platel R. Vesselkin N. P. (1988). Analysis of brain-body allometries in the lamprey *Lampetra fluviatilis* L. J. Evol. Biochem. Phys. 24, 138–145.

[B151] Platel R. Vesselkin N. P. (1989). Comparative study of the encephalization in 3 species of Petromyzonidae (agnatha) - *Petromyzon marinus, Lampetra fluviatilis* and *Lampetra planeri*. J. Hirnforsch. 30, 23–32. 2723409

[B152] Polenova O. A. Vesselkin N. P. (1993). Olfactory and nonolfactory projections in the river lamprey (*Lampetra fluviatilis*) telencephalon. J. Hirnforsch. 34, 261–279. 7693801

[B153] Pollen A. A. Dobberfuhl A. P. Scace J. Igulu M. M. Renn S. C. P. Shumway C. A.. (2007). Environmental complexity and social organization sculpt the brain in Lake Tanganyikan cichlid fish. Brain. Behav. Evol. 70, 21–39. 10.1159/000101067 17389793

[B154] Pombal M. A. Alvarez-Otero R. Perez-Fernandez J. Solveira C. Megias M. (2011). Development and organization of the lamprey telencephalon with special reference to the GABAergic system. Front. Neuroanat. 5:20. 10.3389/fnana.2011.00020 21442003 PMC3062466

[B155] Pombal M. A. Marin O. Gonzalez A. (2001). Distribution of choline acetyltransferase-immunoreactive structures in the lamprey brain. J. Comp. Neurol. 431, 105–126. 10.1002/1096-9861(20010226)431:13.0.CO;2-P 11169993

[B156] Pombal M. A. Megias M. Bardet S. M. Puelles L. (2009). New and old thoughts on the segmental organization of the forebrain in lampreys. Brain Behav. Evol. 74, 7–19. 10.1159/000229009 19729892

[B157] Pombal M. A. Puelles L. (1999). Prosomeric map of the lamprey forebrain based on calretinin immunocytochemistry, Nissl stain, and ancillary markers. J. Comp. Neurol. 414, 391–422. 10516604

[B158] Pombal M. A. Rodicio M. C. Anadon R. (1994). Development and organization of the ocular motor nuclei in the larval sea lamprey, *Petromyzon marinus* L.: an HRP study. J. Comp. Neurol. 341, 393–406. 10.1002/cne.903410309 7515082

[B159] Pombal M. A. Yanez J. Marin O. Gonzalez A. Anadon R. (1999). Cholinergic and GABAergic neuronal elements in the pineal organ of lampreys, and tract-tracing observations of differential connections of pinealofugal neurons. Cell Tissue Res. 295, 215–223. 10.1007/s004410051227 9931367

[B160] Potter I. C. (1980). Ecology of larval and metamorphosing lampreys. Can. J. Fish. Aquat. Sci. 37, 1641–1657. 10.1139/f80-212

[B161] Potter I. C. Gill H. S. Renaud C. B. Haoucher D. (2015). The taxonomy, phylogeny, and distribution of lampreys, in Lampreys: Biology, Conservation and Control, ed Docker M. F. (New York, NY: Springer), 35–73.

[B162] Potter I. C. Hilliard R. W. (1986). Growth and the average duration of larval life in the southern hemisphere lamprey, *Geotria australis* Gray. Experientia 42, 1170–1173. 10.1007/BF01941297

[B163] Potter I. C. Hilliard R. W. Bird D. J. (1980). Metamorphosis in the southern hemisphere lamprey, *Geotria australis*. J. Zool. 190, 405–430. 10.1111/j.1469-7998.1980.tb01435.x

[B164] Potter I. C. Hilliard R. W. Bird D. J. Macey D. J. (1983). Quantitative data on morphology and organ weights during the protracted spawning−run period of the Southern Hemisphere lamprey *Geotria australis*. J. Zool. 200, 1–20. 10.1111/j.1469-7998.1983.tb06106.x

[B165] Potter I. C. Prince P. A. Croxall J. P. (1979). Data on the adult marine and migratory phases in the life cycle of the southern hemisphere lamprey, *Geotria australis* Gray. Env. Biol. Fishes 4, 65–69. 10.1007/BF00005929

[B166] Puzdrowski R. L. Northcutt R. G. (1989). Central projections of the pineal complex in the silver lamprey *Ichthyomyzon unicuspis*. Cell Tissue Res. 255, 269–274. 10.1007/BF00224108 2924332

[B167] Ralph C. L. (1975). The pineal gland and geographical distribution of animals. Int. J. Biometeorol. 19, 289–303. 10.1007/BF01451040 1232070

[B168] Rasband W. S. (1997). ImageJ. Bethesda, MD: U. S. National Institutes of Health 1997–2014. Available online at: http://imagej.nih.gov/ij/

[B169] R Core Team (2013). R: A Language and Environment for Statistical Computing. Vienna: R Foundation for Statistical Computing.

[B170] Reep R. L. Finlay B. L. Darlington R. B. (2007). The limbic system in mammalian brain evolution. Brain Behav. Evol. 70, 57–70. 10.1159/000101491 17409735

[B171] Reis-Santos P. McCormick S. D. Wilson J. M. (2008). Ionoregulatory changes during metamorphosis and salinity exposure of juvenile sea lamprey (*Petromyzon marinus* L.). J. Exp. Biol. 211, 978–988. 10.1242/jeb.014423 18310123

[B172] Ren X. Chang S. Laframboise A. Green W. Dubuc R. Zielinski B. (2009). Projections from the accessory olfactory organ into the medial region of the olfactory bulb in the sea lamprey (*Petromyzon marinus*): a novel vertebrate sensory structure? J. Comp. Neurol. 516, 105–116. 10.1002/cne.22100 19575448

[B173] Renaud C. B. (2011). Lampreys of the World - An Annotated and Illustrated Catalogue of Lamprey Species Known to Date. Rome: Food and Agriculture Organization of the United Nations (FAO).

[B174] Renaud C. B. Gill H. S. Potter I. C. (2009). Relationships between the diets and characteristics of the dentition, buccal glands and velar tentacles of the adults of the parasitic species of lamprey. J. Zool. 278, 231–242. 10.1111/j.1469-7998.2009.00571.x

[B175] Richardson M. K. Admiraal J. Wright G. M. (2010). Developmental anatomy of lampreys. Biol. Rev. 85, 1–33. 10.1111/j.1469-185X.2009.00092.x 19951335

[B176] Robertson B. Saitoh K. Menard A. Grillner S. (2006). Afferents of the lamprey optic tectum with special reference to the GABA input: combined tracing and immunohistochemical study. J. Comp. Neurol. 499, 106–119. 10.1002/cne.21078 16958107

[B177] Ronan M. (1988). Anatomical and physiological evidence for electroreception in larval lampreys. Brain Res. 448, 173–177. 10.1016/0006-8993(88)91115-8 2455583

[B178] Ronan M. Bodznick D. (1991). Behavioral and neurophysiological demonstration of a lateralis skin photosensitivity in larval sea lampreys. J. Exp. Biol. 161, 97–117.

[B179] Ronan M. Northcutt R. G. (1990). Projections ascending from the spinal cord to the brain in petromyzontid and myxinoid agnathans. J. Comp. Neurol. 291, 491–508. 10.1002/cne.902910402 2329187

[B180] Rovainen C. M. (1979). Neurobiology of lampreys. Physiol. Rev. 59, 1007–1077. 227003 10.1152/physrev.1979.59.4.1007

[B181] Rovainen C. M. (1996). Feeding and breathing in lampreys. Brain Behav. Evol. 48, 297–305. 10.1159/000113208 8932870

[B182] Rubinson K. (1990). The developing visual system and metamorphosis in the lamprey. J. Neurobiol. 21, 1123–1135. 10.1002/neu.480210715 2258725

[B183] Saitoh K. Menard A. Grillner S. (2007). Tectal control of locomotion, steering, and eye movements in lamprey. J. Neurophysiol. 97, 3093–3108. 10.1152/jn.00639.2006 17303814

[B184] Sala R. Santamaría C. A. Crespo S. (2005). Growth of organ systems of *Dentex dentex* (L) and *Psetta maxima* (L) during larval development. J. Fish Biol. 66, 315–326. 10.1111/j.0022-1112.2005.00580.x

[B185] Scott W. B. (1887). Notes on the development of Petromyzon. J. Morphol. 1, 253–310. 10.1002/jmor.1050010203

[B186] Siefkes M. J. Scott A. P. Zielinski B. Yun S. S. Li W. (2003). Male sea lampreys, *Petromyzon marinus* L., excrete a sex pheromone from gill epithelia. Biol. Reprod. 69, 125–132. 10.1095/biolreprod.102.014472 12606376

[B187] Smith J. J. Kuraku S. Holt C. Sauka-Spengler T. Jiang N. Campbell M. S.. (2013). Sequencing of the sea lamprey (*Petromyzon marinus*) genome provides insights into vertebrate evolution. Nat. Genet. 45, 415–421. 10.1038/ng.2568 23435085 PMC3709584

[B188] Sorensen P. W. Fine J. M. Dvornikovs V. Jeffrey C. S. Shao F. Wang J.. (2005). Mixture of new sulfated steroids functions as a migratory pheromone in the sea lamprey. Nat. Chem. Biol. 1, 324–328. 10.1038/nchembio739 16408070

[B189] Sousa R. Araújo M. J. Antunes C. (2012). Habitat modifications by sea lampreys (Petromyzon marinus) during the spawning season: effects on sediments. J. Appl. Ichthyol. 28, 766–771. 10.1111/j.1439-0426.2012.02025.x

[B190] Stephan H. (1960). Methodische studien über den quantitativen vergleich architektonischer struktureinheiten des gehirns. Z. Wiss. Zool. 64, 143–172.

[B191] Striedter G. F. (2005). Principles of Brain Evolution. Sunderland, MA: Sinauer Associates, Inc.

[B192] Suárez R. Garcia-Gonzalez D. De Castro F. (2012). Mutual influences between the main olfactory and vomeronasal systems in development and evolution. Front. Neuroanat. 6:50. 10.3389/fnana.2012.00050 23269914 PMC3529325

[B193] Suárez R. Gobius I. Richards L. J. (2014). Evolution and development of interhemispheric connections in the vertebrate forebrain. Front. Hum. Neurosci. 8:497. 10.3389/fnhum.2014.00497 25071525 PMC4094842

[B194] Tamotsu S. Morita Y. (1986). Photoreception in pineal organs of larval and adult lampreys, *Lampetra japonica*. J. Comp. Physiol. 159, 1–5. 10.1007/BF00612489 3746724

[B195] Tomoda H. Uematsu K. (1996). Morphogenesis of the brain in larval and juvenile Japanese eels, *Anguilla japonica*. Brain Behav. Evol. 47, 33–41. 10.1159/000113227 8834783

[B196] Ullen F. Deliagina T. G. Orlovsky G. N. Grillner S. (1995). Spatial orientation in the lamprey. 2. Visual influence on orientation during locomotion and in the attached state. J. Exp. Biol. 198, 675–681. 9318418 10.1242/jeb.198.3.675

[B197] Ullen F. Deliagina T. G. Orlovsky G. N. Grillner S. (1997). Visual pathways for postural control and negative phototaxis in lamprey. J. Neurophysiol. 78, 960–976. 9307127 10.1152/jn.1997.78.2.960

[B198] Vandenbossche J. Seelye J. G. Zielinski B. S. (1995). The morphology of the olfactory epithelium in larval, juvenile and upstream migrant stages of the sea lamprey, *Petromyzon marinus*. Brain Behav. Evol. 45, 19–24. 10.1159/000113382 7866768

[B199] Vernadakis A. J. Bemis W. E. Bittman E. L. (1998). Localization and partial characterization of melatonin receptors in amphioxus, hagfish, lamprey, and skate. Gen. Comp. Endocr. 110, 67–78. 10.1006/gcen.1997.7042 9514841

[B200] Vidal Pizarro I. Swain G. P. Selzer M. E. (2004). Cell proliferation in the lamprey central nervous system. J. Comp. Neurol. 469, 298–310. 10.1002/cne.11013 14694540

[B201] Vigh B. Manzano M. J. Zadori A. Frank C. L. Lukats A. Rohlich P.. (2002). Nonvisual photoreceptors of the deep brain, pineal organs and retina. Histol. Histopathol. 17, 555–590. 11962759 10.14670/HH-17.555

[B202] Villar-Cheda B. Abalo X. M. Villar-Cervino V. Barreiro-Iglesias A. Anadon R. Rodicio M. C. (2008). Late proliferation and photoreceptor differentiation in the transforming lamprey retina. Brain Res. 1201, 60–67. 10.1016/j.brainres.2008.01.077 18295752

[B203] Villar-Cheda B. Perez-Costas E. Melendez-Ferro M. Abalo X. M. Rodriguez-Munoz R. Anadon R.. (2006). Cell proliferation in the forebrain and midbrain of the sea lamprey. J. Comp. Neurol. 494, 986–1006. 10.1002/cne.20851 16385485

[B204] Vrieze L. A. Bergstedt R. A. Sorensen P. W. (2011). Olfactory-mediated stream-finding behavior of migratory adult sea lamprey (*Petromyzon marinus*). Can. J. Fish. Aquat. Sci. 68, 523–533. 10.1139/F10-169

[B205] Vrieze L. A. Bjerselius R. Sorensen P. W. (2010). Importance of the olfactory sense to migratory sea lampreys *Petromyzon marinus* seeking riverine spawning habitat. J. Fish Biol. 76, 949–964. 10.1111/j.1095-8649.2010.02548.x

[B206] Vrieze L. A. Sorensen P. W. (2001). Laboratory assessment of the role of a larval pheromone and natural stream odor in spawning stream localization by migratory sea lamprey (*Petromyzon marinus*). Can. J. Fish. Aquat. Sci. 58, 2374–2385. 10.1139/f01-179

[B207] Wagner C. M. Twohey M. B. Fine J. M. (2009). Conspecific cueing in the sea lamprey: do reproductive migrations consistently follow the most intense larval odour? Anim. Behav. 78, 593–599. 10.1016/j.anbehav.2009.04.027

[B208] Wagner H. J. (2001). Sensory brain areas in mesopelagic fishes. Brain Behav. Evol. 57, 117–133. 10.1159/000047231 11509821

[B209] Wagner H. J. (2003). Volumetric analysis of brain areas indicates a shift in sensory orientation during development in the deep-sea grenadier *Coryphaenoides armatus*. Mar. Biol. 142, 791–797. 10.1007/s00227-002-0990-7

[B210] Wagner H. J. Mattheus U. (2002). Pineal organs in deep demersal fish. Cell Tissue Res. 307, 115–127. 10.1007/s00441-001-0482-y 11810319

[B211] Weigle C. Northcutt R. G. (1998). To the phylogenetic origin of the cerebellum: tracing studies on the silver lamprey *Ichthyomyzon unicuspis*. Eur. J. Neurosci. 10, 196–196.

[B212] Weigle C. Northcutt R. G. (1999). The chemoarchitecture of the forebrain of lampreys: evolutionary implications by comparisons with gnathostomes. Eur. J. Morphol. 37, 122–125. 10.1076/ejom.37.2-3.0122 10342442

[B213] Wicht H. (1996). The brains of lampreys and hagfishes: characteristics, characters, and comparisons. Brain Behav. Evol. 48, 248–261. 10.1159/000113204 8932866

[B214] Willemet R. (2012). Understanding the evolution of mammalian brain structures; the need for a (new) cerebrotype approach. Brain Sci. 2, 203–224. 10.3390/brainsci2020203 24962772 PMC4061787

[B215] Willemet R. (2013). Reconsidering the evolution of brain, cognition, and behavior in birds and mammals. Front. Psychol. 4:396. 10.3389/fpsyg.2013.00396 23847570 PMC3696912

[B216] Yáñez J. Pombal M. A. Anadón R. (1999). Afferent and efferent connections of the parapineal organ in lampreys: a tract tracing and immunocytochemical study. J. Comp. Neurol. 403, 171–189. 9886042 10.1002/(sici)1096-9861(19990111)403:2<171::aid-cne3>3.0.co;2-m

[B217] Yopak K. E. (2012). Neuroecology of cartilaginous fishes: the functional implications of brain scaling. J. Fish Biol. 80, 1968–2023. 10.1111/j.1095-8649.2012.03254.x 22497414

[B218] Yopak K. E. Frank L. R. (2009). Brain size and brain organization of the whale shark, *Rhincodon typus*, using magnetic resonance imaging. Brain Behav. Evol. 74, 121–142. 10.1159/000235962 19729899

[B219] Yopak K. E. Lisney T. J. (2012). Allometric scaling of the optic tectum in cartilaginous fishes. Brain Behav. Evol. 80, 108–126. 10.1159/000339875 22986827

[B220] Yopak K. E. Lisney T. J. Darlington R. B. Collin S. P. Montgomery J. C. Finlay B. L. (2010). A conserved pattern of brain scaling from sharks to primates. Proc. Natl. Acad. Sci. U.S.A. 107, 12946–12951. 10.1073/pnas.1002195107 20616012 PMC2919912

[B221] Yopak K. E. Montgomery J. C. (2008). Brain organization and specialization in deep-sea chondrichthyans. Brain Behav. Evol. 71, 287–304. 10.1159/000127048 18431055

[B222] Yopak K. E. Lisney T. J. Collin S. P. (2015). Not all sharks are “swimming noses”: variation in olfactory bulb size in cartilaginous fishes. Brain Struct. Funct. 220, 1127–1143. 10.1007/s00429-014-0705-0 24435575

[B223] Youson J. H. Wright G. M. Ooi E. C. (1977). The timing of changes in several internal organs during metamorphosis of anadromous larval lamprey *Petromyzon marinus*. Can. J. Zool. 55, 469–473. 10.1139/z77-063 837296

[B224] Zielinski B. Fredricks K. McDonald R. Zaidi A. (2005). Morphological and electrophysiological examination of olfactory sensory neurons during the early developmental prolarval stage of the sea lamprey *Petromyzon marinus* L. J. Neurocytol. 34, 209–216. 10.1007/s11068-005-8354-0 16841164

