# Supplementary material for: Ontogenetic shifts in brain scaling reflect behavioral changes in the life cycle of the pouched lamprey Geotria australis
Source: Front Neurosci. 2015 Jul 28;9:251. doi: 10.3389/fnins.2015.00251 (PMC4517384; doi:10.3389/fnins.2015.00251)
Supplement: Supplementary file 1 [file Table1.DOCX]

***Supplementary Material***

**Ontogenetic shifts in brain scaling reflect behavioral changes in the life cycle of the pouched lamprey *Geotria australis***

**Salas, C. A. ^1^*, Yopak, K. E.^1^, Warrington, R. E.^1^, Hart, N. S.^1^, Potter, I. C.^2^ and Collin, S. P.^1^**

^1^ Neuroecology Group, School of Animal Biology and UWA Oceans Institute, University of Western Australia, Crawley, WA, Australia

^2^ Centre for Fish and Fisheries Research, School of Veterinary and Life Sciences, Murdoch University, Murdoch, WA, Australia

*** Correspondence:** Mr. Carlos Salas, The University of Western Australia, School of Animal Biology, Neuroecology Group, 35 Stirling Highway, Crawley, WA, 6009, Australia

[carlos.salas.uwa](mailto:carlos.salas.uwa)@gmail.com

**Supplementary Table 1. Summary of the brain data**. Average ± standard deviation of the parameters measured for each stage. (*) Body mass averages from 2 specimens of fourth age class ammocoetes (amIV) and 5 specimens of upstream migrants (us). (**) Brain subdivisions averages calculated from 8 specimens. OB: olfactory bulbs, Te: telencephalic hemispheres, PO: pineal organ, OT: optic tectum, OCT: octaval-trigeminal region, GUS: gustatory region; PFA: paraformaldehyde, PBS: phosphate buffer. amII: second age class ammocoetes, amIII: third age class ammocoetes, ds: downstream migrants, us: upstream migrants, sa: spawning adults.

| **stage**  **abbrev** | **n** | **fixation/preservation**  **methods** | **body mass**  (g) | **brain mass**  (mg) | **OB**  (μL) | **Te**  (μL) | **PO**  (μL) | **OT**  (μL) | **OCT**  (μL) | **GUS**  (μL) |
| --- | --- | --- | --- | --- | --- | --- | --- | --- | --- | --- |
| amII | 6 | Bouins / ethanol | 0.32±  0.03 | 0.62±  0.08 | 0.03±  0.01 | 0.03±  4.47E-03 | 4.43E-03±  1.84E-03 | 0.04±  0.01 | 0.10±  0.02 | 0.21±  0.03 |
| amIII | 5 | Bouins / ethanol | 1.03±  0.18 | 1.00±  0.18 | 0.05±  0.01 | 0.05±  9.26E-03 | 9.80E-03±  4.32E-03 | 0.05±  0.01 | 0.21±  0.05 | 0.26±  0.04 |
| amIV | 3 * | Bouins / ethanol | 1.92±  0.42 | 1.45±  0.35 | 0.07±  0.01 | 0.07±  7.09E-02 | 1.63E-02±  1.63E-02 | 0.04±  0.01 | 0.28±  0.05 | 0.36±  0.03 |
| ds | 6 | PFA / ethanol | 1.33±  0.28 | 5.69±  0.34 | 0.40±  0.08 | 0.48±  1.41E-01 | 1.48E-02±  6.31E-03 | 1.21±  0.06 | 0.61±  0.08 | 0.69±  0.15 |
| us | 11 * | PFA / PBS | 180.28±  28.59 | 27.60±  0.55 | 2.78±  0.41 | 1.22±  1.96E-01 | 5.48E-02±  1.42E-02 | 2.93±  0.84 | 4.12±  0.51 | 4.49±  0.61 |
| sa | 9 ** | PFA / ethanol | 133.36±  22.16 | 20.00±  5.62 | 2.12±  0.83 | 1.26±  5.77E-01 | 6.74E-02±  3.21E-02 | 2.01±  0.74 | 3.57±  0.92 | 2.80±  0.62 |
