# Supplementary material for: Ontogenetic shifts in brain scaling reflect behavioral changes in the life cycle of the pouched lamprey Geotria australis
Source: Front Neurosci. 2015 Jul 28;9:251. doi: 10.3389/fnins.2015.00251 (PMC4517384; doi:10.3389/fnins.2015.00251)
Supplement: Supplementary file 2 [file Table2.DOCX]

***Supplementary Material***

**Ontogenetic shifts in brain scaling reflect behavioral changes in the life cycle of the pouched lamprey *Geotria australis***

**Salas, C. A. ^1^*, Yopak, K. E.^1^, Warrington, R. E.^1^, Hart, N. S.^1^, Potter, I. C.^2^ and Collin, S. P.^1^**

^1^ Neuroecology Group, School of Animal Biology and UWA Oceans Institute, University of Western Australia, Crawley, WA, Australia

^2^ Centre for Fish and Fisheries Research, School of Veterinary and Life Sciences, Murdoch University, Murdoch, WA, Australia

*** Correspondence:** Mr. Carlos Salas, The University of Western Australia, School of Animal Biology, Neuroecology Group, 35 Stirling Highway, Crawley, WA, 6009, Australia

[carlos.salas.uwa](mailto:carlos.salas.uwa)@gmail.com

**Supplementary Table 2. Grouping of stages for each of the factors modeled in the ANCOVA analyses.** See text for more details. amII: second age class ammocoetes, amIII: third age class ammocoetes, am IV: fourth age class ammocoetes, ds: downstream migrants, us: upstream migrants, sa: spawning adults.

| **Factor** | **Stages** | | | | | | |
| --- | --- | --- | --- | --- | --- | --- | --- |
|  | **amII** | **amIII** | **amIV** | **ds** | **us** | **sa** | **n** |
| stage 1 | amII | amIII | amIV | ds | us | sa | 6 |
| stage 2 | ammocoetes | | | ds | us | sa | 4 |
| stage 3 | amII | amIII | amIV | all adults | | | 4 |
| stage 4 | ammocoetes | | | ds + us | | sa | 3 |
| stage 5 | ammocoetes | | | ds | us + sa | | 3 |
| stage 6 | ammocoetes | | | all adults | | | 2 |
