# Supplementary material for: Ontogenetic shifts in brain scaling reflect behavioral changes in the life cycle of the pouched lamprey Geotria australis
Source: Front Neurosci. 2015 Jul 28;9:251. doi: 10.3389/fnins.2015.00251 (PMC4517384; doi:10.3389/fnins.2015.00251)
Supplement: Supplementary file 3 [file Table3.DOCX]

***Supplementary Material***

**Ontogenetic shifts in brain scaling reflect behavioral changes in the life cycle of the pouched lamprey *Geotria australis***

**Salas, C. A. ^1^*, Yopak, K. E.^1^, Warrington, R. E.^1^, Hart, N. S.^1^, Potter, I. C.^2^ and Collin, S. P.^1^**

^1^ Neuroecology Group, School of Animal Biology and UWA Oceans Institute, University of Western Australia, Crawley, WA, Australia

^2^ Centre for Fish and Fisheries Research, School of Veterinary and Life Sciences, Murdoch University, Murdoch, WA, Australia

*** Correspondence:** Mr. Carlos Salas, The University of Western Australia, School of Animal Biology, Neuroecology Group, 35 Stirling Highway, Crawley, WA, 6009, Australia

[carlos.salas.uwa](mailto:carlos.salas.uwa)@gmail.com

**Supplementary Table 3. Summary of model selection**. Values of the second-order Akaike information criterion (AICc) and the difference of this value with the selected model (ΔAICc) are given below. (*) ΔAICc < 2, models were selected using model average (see methods), (**) linear model assumptions were violated, (***) the volume of the telencephalic vesicles is compared to the volume of the olfactory bulbs (see results). OB: olfactory bulbs, Te: telencephalic hemispheres, PO: pineal organ, OT: optic tectum, OCT: octaval-trigeminal region, GUS: gustatory region.

| Factor | Linear models | | | | | | | | | | | | | |
| --- | --- | --- | --- | --- | --- | --- | --- | --- | --- | --- | --- | --- | --- | --- |
|  | brain to body mass | | brain subdivision to total brain minus brain subdivision volume | | | | | | | | | | | |
|  |  |  | OB | | Te*** | | PO | | OT | | OCT | | GUS | |
|  | AICc | ΔAICc | AICc | ΔAICc | AICc | ΔAICc | AICc | ΔAICc | AICc | ΔAICc | AICc | ΔAICc | AICc | ΔAICc |
| no factor | ** | ** | -59.79 | 0* | ** | ** | -16.48 | 8.44 | ** | ** | -41.79 | 34.24 | ** | ** |
| stage 1 | -79.84 | 6.69 | -55.32 | 4.47 | -76.13 | 6.76 | -22.22 | 2.70 | -53.28 | 2.38 | -76.03 | 0 | -73.20 | 2.54 |
| stage 2 | -86.53 | 0 | -56.91 | 2.88 | -82.19 | 0.71* | -24.92 | 0* | -54.85 | 0.81* | -64.05 | 11.98 | -75.74 | 0* |
| stage 3 | ** | ** | -52.54 | 7.25 | -79.85 | 3.04 | -17.66 | 7.26 | -51.88 | 3.78 | -51.77 | 24.26 | -62.16 | 13.58 |
| stage 4 | -81.12 | 5.41 | -56.88 | 2.91 | -80.64 | 2.25 | -24.76 | 0.16* | -55.66 | 0* | -62.80 | 13.23 | ** | ** |
| stage 5 | -81.24 | 5.29 | -59.69 | 0.10* | -82.61 | 0.28* | -16.32 | 8.60 | -49.66 | 6.00 | ** | ** | -73.76 | 1.98* |
| stage 6 | ** | ** | -57.95 | 1.83* | -82.89 | 0* | -18.92 | 6.00 | -52.29 | 3.37 | -54.67 | 21.36 | ** | ** |
