# Supplementary material for: Ontogenetic shifts in brain scaling reflect behavioral changes in the life cycle of the pouched lamprey Geotria australis
Source: Front Neurosci. 2015 Jul 28;9:251. doi: 10.3389/fnins.2015.00251 (PMC4517384; doi:10.3389/fnins.2015.00251)
Supplement: Supplementary file 4 [file Table4.DOCX]

***Supplementary Material***

**Ontogenetic shifts in brain scaling reflect behavioral changes in the life cycle of the pouched lamprey *Geotria australis***

**Salas, C. A. ^1^*, Yopak, K. E.^1^, Warrington, R. E.^1^, Hart, N. S.^1^, Potter, I. C.^2^ and Collin, S. P.^1^**

^1^ Neuroecology Group, School of Animal Biology and UWA Oceans Institute, University of Western Australia, Crawley, WA, Australia

^2^ Centre for Fish and Fisheries Research, School of Veterinary and Life Sciences, Murdoch University, Murdoch, WA, Australia

*** Correspondence:** Mr. Carlos Salas, The University of Western Australia, School of Animal Biology, Neuroecology Group, 35 Stirling Highway, Crawley, WA, 6009, Australia

[carlos.salas.uwa](mailto:carlos.salas.uwa)@gmail.com

**Supplementary Table 4. Summary of the parameters of the linear models of brain mass as a function of body mass**. Plots of these equations are shown in Figure 4. For further details see Methods. (***) p-value < 0.001, (**) 0.001 < p-value < 0.01, (*) 0.01 < p-value < 0.05, (.) 0.05 < p-value < 0.1, ( ) p-value > 0.1.

| **linear model** | **factor** | **n** | **stage**  **abbrev** | **intercept** | **slope** | **R-squared** | **p-value** | **global stats** |
| --- | --- | --- | --- | --- | --- | --- | --- | --- |
| **ontogenetic**  **regression** | none | 32 | all  stages | -0.8855 | 0.52619 | 0.8568 | - | NO |
| **stage-specific**  **regressions** | none | 13 | am | -0.8656 (***) | 0.47135 (***) | 0.9031 | 4.025e-07 | OK |
|  |  | 6 | ds | 0.9631 (.) | 0.1912 | 0.3260 | 0.1382 | OK |
|  |  | 5 | us | 1.8826 (*) | 0.08929 | 0.3169 | 0.1896 | OK |
|  |  | 8 | sa | -3.2174 | 0.8971 (.) | 0.3455 | 0.0562 | OK |
| **ANCOVA** | stage 2 | 13 | am | -0.8572 (***) | 0.46915 (***) | 0.9927 | < 2.2e-16 | OK |
|  |  | 6 | ds | -0.1823 (***) |  |  |  |  |
|  |  | 5 | us | -0.4921(**) |  |  |  |  |
|  |  | 8 | sa | -0.5945 (*) |  |  |  |  |
