# Supplementary material for: Ontogenetic shifts in brain scaling reflect behavioral changes in the life cycle of the pouched lamprey Geotria australis
Source: Front Neurosci. 2015 Jul 28;9:251. doi: 10.3389/fnins.2015.00251 (PMC4517384; doi:10.3389/fnins.2015.00251)
Supplement: Supplementary file 5 [file Table5.DOCX]

***Supplementary Material***

**Ontogenetic shifts in brain scaling reflect behavioral changes in the life cycle of the pouched lamprey *Geotria australis***

**Salas, C. A. ^1^*, Yopak, K. E.^1^, Warrington, R. E.^1^, Hart, N. S.^1^, Potter, I. C.^2^ and Collin, S. P.^1^**

^1^ Neuroecology Group, School of Animal Biology and UWA Oceans Institute, University of Western Australia, Crawley, WA, Australia

^2^ Centre for Fish and Fisheries Research, School of Veterinary and Life Sciences, Murdoch University, Murdoch, WA, Australia

*** Correspondence:** Mr. Carlos Salas, The University of Western Australia, School of Animal Biology, Neuroecology Group, 35 Stirling Highway, Crawley, WA, 6009, Australia

[carlos.salas.uwa](mailto:carlos.salas.uwa)@gmail.com

**Supplementary Table 5. Summary of the parameters of the linear models of brain subdivisions volumes as a function of total brain volume minus brain subdivision volume**. Plots of the best model are shown in Figure 5. (+)Telencephalic hemispheres as a function of total volume of the olfactory bulbs. For further details see Methods and Results.

| **linear model** | **structure** | **factor** | **n** | **stage** | **intercept** | **slope** | **R-squared** | **p-value** | **global stats** |
| --- | --- | --- | --- | --- | --- | --- | --- | --- | --- |
| **Ontogenetic**  **regression** | OB | none | 39 | all  stages | -2.12 (***) | 1.27 (***) | 0.984 | < 2.2e-16 | OK |
|  | Te |  |  |  | -1.27 | 1.01 | 0.963 | - | NO |
|  | PO |  |  |  | -1.04 (***) | 0.63 (***) | 0.838 | < 2.2e-16 | OK |
|  | OT |  |  |  | -1.88 | 1.24 | 0.905 | - | NO |
|  | OCT |  |  |  | -0.53 (***) | 0.95 (***) | 0.958 | < 2.2e-16 | OK |
|  | GUS |  |  |  | 0.35 | 0.73 | 0.947 | - | NO |
| **Best model** | OB | none | 39 | all stages | -2.12 (***) | 1.27 (***) | 0.984 | < 2.2e-16 | OK |
|  | Te+ | stage 6 | 14 | am | -0.32 (***) | 0.64 (***) | 0.987 | < 2.2e-16 | OK |
|  |  |  | 25 | adults | 0.62 (***) |  |  |  |  |
|  | PO | stage 2 | 14 | am | -2.54 (***) | 1.16 (***) | 0.884 | 2.877e-16 | OK |
|  |  |  | 6 | ds | -3.13 (**) |  |  |  |  |
|  |  |  | 11 | us | -3.41 (*) |  |  |  |  |
|  |  |  | 8 | sa | -3.14 (.) |  |  |  |  |
|  | OT | stage 4 | 14 | am | 2.23(***) | 0.47 (***) | 0.983 | < 2.2e-16 | OK |
|  |  |  | 17 | ds+us | 3.40 (***) |  |  |  |  |
|  |  |  | 8 | sa | 3.28 (*) |  |  |  |  |
|  | OCT | stage 1 | 6 | amII | 0.88 (.) | 0.41 (*) | 0.986 | < 2.2e-16 | OK |
|  |  |  | 5 | amIII | 1.15 (***) |  |  |  |  |
|  |  |  | 3 | amIV | 1.23 (***) |  |  |  |  |
|  |  |  | 6 | ds | 1.27 (*) |  |  |  |  |
|  |  |  | 11 | us | 1.84 (**) |  |  |  |  |
|  |  |  | 8 | sa | 1.83 (**) |  |  |  |  |
|  | GUS | stage 2 | 14 | am | 1.20 (***) | 0.43 (***) | 0.977 | < 2.2e-16 | OK |
|  |  |  | 6 | ds | 1.25 |  |  |  |  |
|  |  |  | 11 | us | 1.70 (**) |  |  |  |  |
|  |  |  | 8 | sa | 1.62 (**) |  |  |  |  |
